# Transcutaneous Electrical Nerve Stimulation and Pain With Movement in People With Fibromyalgia: A Cluster Randomized Clinical Trial

**DOI:** 10.1001/jamanetworkopen.2026.2450

**Published:** 2026-03-27

**Authors:** Dana L. Dailey, Carol G. T. Vance, Barbara J. Van Gorp, Elizabeth M. Johnson, Andrew A. Post, Ruth L. Chimenti, Kari G. Vance, Carla Franck, Josiah Sault, Ezgi Yarasir, Heather S. Reisinger, Alexandra Anderson, Jesse Anderson, Randy Capelle, Amanda Crouch, Jeffrey Donatelle, Dennis Kaster, Ted Kepros, Emily Nicklies, Bill Rein, Robert Worth, Mariah Balinski, David-Erick Lafontant, E. J. Slade, Fangfang Jiang, Dixie Ecklund, Tina Neill-Hudson, Maxine Koepp, Michele Costigan, Maggie Spencer, Kristin R. Archer, Bridget M. Zimmerman, Emine O. Bayman, Leslie J. Crofford, Kathleen A. Sluka

**Affiliations:** 1 Department of Physical Therapy and Rehabilitation Science, University of Iowa, Iowa City; 2 Department of Physical Therapy, St Ambrose University, Davenport, Iowa; 3 University of Iowa Health Care, North Liberty; 4 Division of Rheumatology, Vanderbilt University Medical Center, Nashville, Tennessee; 5 Department of Physical Therapy, Grand Valley State University, Allendale, Michigan; 6 Kepros Physical Therapy and Performance, Cedar Rapids, Iowa; 7 UI Health, University of Illinois Chicago, Chicago; 8 Department of Internal Medicine, University of Iowa, Iowa City; 9 Big Stone Therapies, Baxter, Minnesota; 10 Rock Valley Physical Therapy, Muscatine, Iowa; 11 Advanced Physical Therapy & Sports Medicine, Stevens Point, Wisconsin; 12 Advanced Physical Therapy & Sports Medicine, Appleton, Wisconsin; 13 Department of Biostatistics, College of Public Health, University of Iowa, Iowa City; 14 Department of Orthopaedic Surgery, Vanderbilt University Medical Center, Nashville, Tennessee

## Abstract

**Importance:**

Fibromyalgia is characterized by chronic widespread pain that is often exacerbated by movement that interferes with daily activities. Development of effective treatments for movement-evoked pain is essential for improving function for individuals with fibromyalgia.

**Objective:**

To evaluate whether the addition of transcutaneous electrical nerve stimulation (TENS) to outpatient physical therapy improves fibromyalgia-associated movement-evoked pain.

**Design, Setting, and Participants:**

The Fibromyalgia TENS in Physical Therapy (FM-TIPS) study was a cluster-randomized clinical trial of participants with fibromyalgia at 28 outpatient PT clinics from 6 health care systems. Between February 1, 2021, and September 31, 2024, 958 participants were screened, 459 participants enrolled, and 384 completed baseline data collection, with final data collected in March 2025.

**Intervention:**

Clinics were randomized to PT plus TENS (PT-TENS) and PT-only groups. Data were captured on days 1, 30, 60 (primary end point, randomized phase), 90, and 180. Participants in the PT-only group received TENS after day 60 (extension phase). TENS was applied to the upper and lower back with instructions to use 2 hours daily with parameters of modulating frequency of 2 to 125 Hz for 100 to 180 microseconds at a strong but comfortable intensity.

**Main Outcomes and Measures:**

The primary outcome was a change in movement-evoked pain (scale of 0-10, with 0 indicating no pain and 10 indicating worst pain imaginable) from baseline to day 60 rated during a 5-times sit-and-stand task using a linear mixed-effects model. In addition, patient-reported improvement based on the Patient Global Impression of Change score and patient-reported adverse events were assessed.

**Results:**

A total of 384 FM-TIPS participants (mean [SD] age, 53 [15] years; 351 [91%] female) completed baseline data collection (modified intention-to-treat), with 191 individuals in PT-TENS group and 193 in PT-only group. Movement-evoked pain at day 60 during TENS treatment was significantly lower in the PT-TENS group compared with the PT-only group (group mean difference, −1.2; 95 CI, −1.6 to −0.7; *d* = 0.46). A dose-response effect for TENS was observed, with more participants in the PT-TENS group reporting improvement on the Patient Global Impression of Change (120 [72%] vs 86 [51%], *P* = .001) and a 30% or greater reduction in movement-evoked pain in responder analysis (66 of 161 [41%] vs 22 of 169 [13%]; *P* < .001). At day 180, 217 respondents (81%) found TENS helpful and 147 (55%) used TENS daily. There were no serious adverse events, and 109 of 358 (30%) experienced minor adverse events during the entire 6 months of the study.

**Conclusions and Relevance:**

In this cluster randomized clinical trial of TENS in fibromyalgia, TENS meaningfully reduced movement-evoked pain and remained effective for 6 months. This study’s results suggest that TENS is a safe, inexpensive, and readily available treatment for fibromyalgia.

**Trial Registration:**

ClinicalTrials.gov Identifier: NCT04683042

## Key Points

**Question:**

Is transcutaneous electrical nerve stimulation (TENS) effective for treating pain when combined with physical therapy (PT) in individuals with fibromyalgia?

**Findings:**

This cluster randomized clinical trial of 459 participants with fibromyalgia from 28 PT clinics from 6 health systems found that adding TENS to routine PT resulted in a statistically significant and clinically meaningful reduction of movement-evoked pain at 2 months, with effectiveness sustained for at least 6 months.

**Meaning:**

This study’s findings suggest that TENS is a safe and effective modality for managing fibromyalgia pain.

## Teaser

This cluster randomized clinical trial examines whether transcutaneous electrical nerve stimulation paired with routine physical therapy vs physical therapy alone reduces movement-evoked pain in individuals with fibromyalgia.

## Introduction

Fibromyalgia is a complex condition characterized by chronic widespread pain and accompanied by fatigue, nonrefreshing sleep, and cognitive dysfunction.^1,2,3^ Fibromyalgia pain is exacerbated with movement that contributes to reduced function.^3^ Treatment guidelines recommend nonpharmacologic approaches with exercise as a first-line intervention.^4,5,6,7,8,9,10,11,12,13,14,15,16,17^ However, adherence to exercise is often poor due to movement-evoked pain, which presents a significant barrier to participation.^18,19,20^

Transcutaneous electrical nerve stimulation (TENS) is a safe, inexpensive, nonpharmacological treatment often used by physical therapists to reduce pain in a variety of conditions, including fibromyalgia.^21,22^ Mechanistically, TENS activates endogenous inhibitory mechanisms in the central nervous system that subsequently reduce central excitability in pain transmission pathways.^21^ Thus, TENS may be particularly useful for individuals with altered central pain processing.

Although the mechanisms underlying fibromyalgia are heterogeneous, there is strong evidence of altered central pain processing, making fibromyalgia a candidate condition to manage with TENS. A prior placebo-controlled randomized clinical trial (Fibromyalgia Activity Study With TENS [FAST])^23^ observed improvements in movement-evoked pain during TENS compared with placebo or no TENS in women with fibromyalgia. However, FAST used a selected clinical population that excluded individuals with potential confounding comorbidities, was conducted in a research setting, and tested efficacy for only 1 month.

The Fibromyalgia TENS in Physical Therapy Study (FM-TIPS) was a pragmatic cluster randomized clinical trial designed to test effectiveness of TENS in a clinical setting for 6 months. This trial was pragmatic as it was embedded into physical therapy (PT) clinics during routine care; local clinicians screened, enrolled, and provided the intervention; and all individuals with fibromyalgia were included as long as they did not have a contraindication to use of the intervention. We tested whether TENS paired with routine PT (PT-TENS group) would reduce movement-evoked pain in individuals with fibromyalgia when compared with PT alone (PT-only group).

## Methods

### Study Design

FM-TIPS was a pragmatic cluster randomized clinical trial approved by the University of Iowa institutional review board. Participants were enrolled after completion of electronic informed consent between February 1, 2021, and September 31, 2024, with final data collected in March 2025. A detailed study protocol has been published^24^ and is available in Supplement 1. Additional methodology is available in the eMethods in Supplement 2. The study followed the Consolidated Standards of Reporting Trials (CONSORT) reporting guideline.

### Study Participants

Twenty-eight clinics screened and enrolled participants. Demographic data, including race and ethnicity, were collected to characterize the sample. Race categories included African American or Black, American Indian or Alaska Native, Asian, Native Hawaiian or Pacific Islander, White, multiracial, and unknown or missing; ethnicity categories included Hispanic or Latino, not Hispanic or Latino, and unknown or missing. Clinics were randomized by a study statistician (D.-E.L., B.M.Z., or E.O.B.) to the PT-TENS (n = 13) or PT-only (n = 15) group and stratified by health care system and clinic size (large [>3 physical therapists] or small [≤3 physical therapists]).

**Figure 1.**
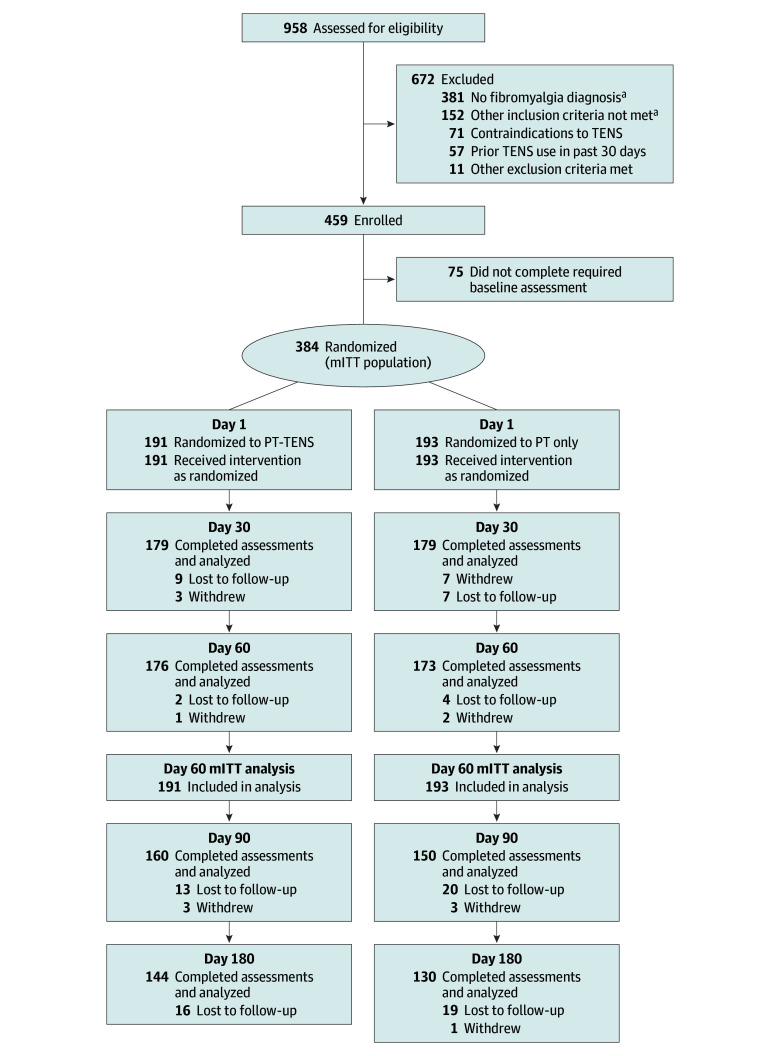
CONSORT Flow Diagram We randomized 384 participants to clinics providing physical therapy (PT) and transcutaneous electrical nerve stimulation (TENS) (PT-TENS group) or PT only (PT-only group) who served as our modified intention-to-treat (mITT) population by completing baseline (day 1) assessments. After day 60 (end of randomized phase and primary end point), the PT-TENS group continued using TENS, and the PT-only group was provided TENS units and telehealth instructions to use through day 180 (extension phase). A total of 79 physical therapists were trained and participated in the PT-TENS group and 82 in the PT-only group. ^a^Eligibility failure could occur for more than one reason.

A total of 958 participants were screened, 459 participants enrolled, and 384 completed baseline data collection (modified intention to treat [mITT]). There were 191 participants in the PT-TENS group and 193 in the PT-only group (Figure 1). All participants received the intervention as assigned and none were excluded from the mITT analysis. After completion of the day 60 assessments, 176 participants in the PT-TENS group continued TENS and 173 participants in the PT-only group received TENS (extension phase). A total of 144 participants in the PT-TENS group and 130 in the PT-only group completed day 180.

#### Study Flow

Study participation lasted 6 months. During the randomized phase (days 1-60), both groups received routine PT as recommended by their physical therapist. In the extension phase (days 60-180), both groups used TENS. The PT-only group was mailed a TENS unit and received virtual instruction after completing day 60. Assessments were completed remotely with Research Electronic Data Capture (REDCap) on days 1, 30, 60 (primary outcome), 90, and 180.

Participants completed screening, enrollment, and study training during their first 3 PT visits. On PT visit 1, patients presenting with chronic pain and/or fibromyalgia were assessed for eligibility with an electronic screening survey (REDCap). Adults (aged >18 years) were included in the study if diagnosed with fibromyalgia by a clinician, able to read English, and willing to use TENS. Patients were excluded if they had contraindications to TENS or TENS use in the last 30 days. On PT visit 2, participants received study materials and instructions on TENS use and/or study procedures by their physical therapist. Participants were asked to complete baseline data collection at home before PT visit 3.

Each assessment session consisted of resting pain and fatigue ratings and a 5 times repeated sit-and-stand test with movement-evoked pain and movement-evoked fatigue ratings. The PT-TENS group then applied TENS and the PT-only rested quietly while completing additional surveys. Next, participants repeated the sit-and-stand test with movement-evoked pain and fatigue ratings with after 30 minutes of TENS with the units active, if applicable.

### Intervention

TENS (NeuroMetrix Inc) was applied through electrodes (butterfly 4 × 6 in, CompassHealth) placed on the upper and lower back. Parameters were an asymmetric biphasic waveform with a modulating frequency of 2 to 125 Hz for a pulse duration of 100 to 180 microseconds and a strong but comfortable stimulation intensity. Participants were instructed to wear TENS for 2 hours daily during activity with a minimum of 30 minutes per session. Use was monitored with a custom nerve stimulation device (Quell Flex, Quell Wearable Pain Relief Technology) that recorded data on a cloud-based server. Participants were instructed to open the application once a week, which uploaded use data to a cloud.

### Outcomes

Demographics, baseline data and medication use were collected by self-report. The primary outcome measure was change in movement-evoked pain from baseline before TENS to day 60 after TENS measured on a scale of 0 to 10, with 0 indicating no pain and 10 indicating worst pain imaginable. The sit-and-stand test used in the prior randomized clinical trial^23^ was adapted for FM-TIPS to be completed by participants at home because of an observed clinically significant reduction in movement-evoked pain with TENS.^23^ Secondary and exploratory outcome measures included additional measures of pain, fatigue, function, disease severity, and HEAL (Helping to End Addiction Long-Term) common data elements. These measures are further described in the published protocol article for FM-TIPS.^24^In addition, self-report adverse events and Patient Global Impression of Change scores were collected at all time points after baseline. Participants completed a survey asking about their experience with TENS on day 180.

### Sample Size Calculation

To reach 80% power with a 2-sided type 1 error rate of 0.05, the original sample size was estimated at 600 enrolled patients. This was based on detecting a mean (SD) difference of 1.0 (2.0) between the PT-TENS and the PT-only groups for change in movement-evoked pain. We used an intraclass correlation coefficient (ICC) of 0.12 for 25 participants per clinic for 24 clinics. A planned interim reassessment of the ICC was performed after the first 200 participants completed day 60 assessments. The target sample size was recalculated assuming an observed ICC of 0.10 and a coefficient of variation of 0.60 for the number of patients enrolled per clinic. Sample size was reduced to 450, leaving us with an estimate of 342 participants completing day 60 based on a 24% dropout rate. The final ICC was calculated as 0.01, calculated using a generalized linear mixed model with type I sums of squares.

### Statistical Analysis

Following the prespecified analysis plan (Supplement 1), an mITT plan was completed for all participants with complete baseline data. Linear mixed-effects models analyzed differences in the change from baseline to day 60 between the PT-TENS and the PT-only groups. Movement-evoked pain, resting pain, movement-evoked fatigue, and resting fatigue were calculated as a change from day 1 before TENS to day 60 after TENS. Secondary and exploratory outcomes were calculated as change from day 1 to day 60. These models included random effects for treatment nested within the clinic and health care systems and fixed effects for treatment group, clinic size, and baseline values. Model estimation was conducted using residual maximum likelihood. Sensitivity analyses were conducted to evaluate the robustness of results under different missing data mechanisms, for fibromyalgia-positive vs fibromyalgia-negative groups, and for analysis of the Rapid Assessment of Physical Activity questionnaire (Supplement 2). Treatment effects were tested using 2-sided tests at a *P* < .05 significance level and summarized as means with 95% CIs. The Cohen *d* estimated effect sizes. Per protocol analysis defined a minimally effective dose (8 times per month, 1600 minutes per month) based on the prior study.^23^ The number of responders on day 60 was calculated based on prior studies showing a clinically important difference of 30% or more in movement-evoked pain^25^ and resting pain.^26,27^

## Results

### Participants

A total of 384 FM-TIPS participants (mean [SD] age, 53 [15] years; 351 [91%] female; 32 [8%] African American or Black; 3 [<1%] American Indian or Alaska Native, 3 [<1%] Asian, 2 [<1%] Native Hawaiian or Pacific Islander, 315 [82%], 7 [2%] multiracial, 26 [7%] unknown or missing; 26 [2%] Hispanic or Latino, 326 [85%] not Hispanic or Latino, 22 [6%] unknown or missing) completed baseline data collection (modified intention-to-treat), with 191 individuals in PT-TENS group and 193 in PT-only group. Participants experienced moderate resting pain (mean [SD] score, 5.4 [2.0]) and fatigue (mean [SD] score, 5.9 [2.3]), with a mean (SD) Fibromyalgia Impact Questionnaire Revised (FIQR) score of 57.4 (17.4). A total of 296 participants (74%) met the American College of Rheumatology 2016 criteria for fibromyalgia at baseline. Additional demographic and baseline measures are given in Table 1 and eTable 1 in Supplement 2.

**Table 1.** Baseline Characteristics of the Study Participants

Characteristic	No. (%) of participants^a^		
	Total (N = 384)	PT-TENS (n = 191)	PT only (n = 193)
Age, mean (SD), y	53.2 (15.2)	52.6 (15.5)	53.9 (14.8)
Sex			
Female	351 (91)	175 (92)	176 (9)
Male	33 (9)	16 (8)	17 (9)
Ethnicity			
Hispanic or Latino	26 (2)	7 (4)	19 (10)
Not Hispanic or Latino	326 (85)	170 (89)	156 (81)
Unknown or missing	22 (6)	9 (5)	13 (7)
Race			
African American or Black	32 (8)	17 (9)	15 (8)
American Indian or Alaska Native	3 (<1)	1 (<1)	2 (1)
Asian	3 (<1)	3 (2)	9 (0)
Native Hawaiian or Pacific Islander	2 (<1)	0	2 (1)
White	315 (82)	156 (82)	159 (82)
Multiracial	7 (2)	3 (2)	4 (2)
Unknown or missing	26 (7)	11 (6)	11 (6)
Educational level			
Postsecondary	224 (60)	114 (61)	110 (59)
High school or less	149 (40)	73 (39)	76 (41)
Missing	11 (3)	4 (2)	7 (4)
Yearly income, $			
>50 000	159 (48)	84 (51)	75 (46)
<50 000	171 (52)	82 (49)	89 (54)
Environment			
Rural (RUCA code 4-10)	193 (50)	79 (41)	114 (59)
Urban (RUCA code 1-3)	191 (50)	112 (59)	79 (41)
Employment			
Employed	160 (43)	84 (45)	76 (41)
Unemployed	210 (57)	102 (55)	108 (59)
Missing	14 (4)	5 (3)	9 (5)
Disability insurance	107 (28)	51 (27)	56 (29)
Relationship status			
Married	238 (64)	117 (64)	121 (64)
Unmarried	135 (36)	66 (36)	69 (36)
Missing	11 (3)	8 (4)	3 (2)
Fibromyalgia measures			
Fibromyalgia impact (FIQR, scale of 0-100) score, mean (SD)^b^	57.4 (17.4)	57.5 (17.3)	57.2 (17.5)
Widespread pain score (WPI, scale of 0-19), mean (SD)^b^	9.3 (4.0)	9.2 (4.1)	9.4 (4.0)
Somatic symptoms score (SSS, scale of 0-12), mean (SD)^b^	7.5 (2.3)	7.5 (2.4)	7.5 (2.1)
PSD score (scale of 0-31), mean (SD)^b^	16.9 (5.3)	16.8 (5.6)	17.1 (5.0)
Fibromyalgia positive^c^	286 (74)	139 (73)	147 (76)
Pain duration, median (IQR), y^b^	11.2 (5.9-20.5)	11.0 (5.9-20.0)	11.4 (5.9-21.0)
Baseline measures of outcome variables, mean (SD)			
MEP (NRS, scale of 0-10)^b^	5.6 (2.2)	5.5 (2.1)	5.6 (2.3)
Pain at rest (NRS, scale of 0-10)^b^	5.4 (2.0)	5.5 (2.0)	5.9 (1.7)
Pain interference (BPI, scale of 0-10)^b^	6.1 (2.1)	6.2 (2.1)	6.0 (2.1)
Pain severity (BPI, scale of 0-10)^b^	5.9 (1.7)	5.8 (1.8)	5.9 (1.7)
Global Fatigue Index (MAF, scale of 0-10)^b^	33.9 (9.3)	34.2 (9.1)	33.7 (9.6)
Fatigue at rest (NRS, scale of 0-10)^b^	5.9 (2.3)	5.8 (2.3)	5.9 (2.3)
Fatigue with movement (NRS, scale of 0-10)^b^	6.0 (2.3)	6.0 (2.3)	6.0 (2.2)
Sleep (PROMIS, T score)^b^	56.3 (4.2)	56.3 (4.1)	56.2 (4.3)
Sleep duration (PROMIS, hours)^b^	6.2 (1.8)	6.2 (1.8)	6.2 (1.8)
Pain catastrophizing (PCS, scale of 0-52)^b^	23.6 (14.2)	23.8 (14.5)	23.5 (13.8)
Depression (PHQ-8, scale of 0-24)^b^	10.8 (5.6)	11.0 (6.0)	10.5 (5.2)
Anxiety (GAD-7, scale of 0-21)^b^	8.0 (5.9)	8.1 (6.2)	7.9 (5.6)
Activity average (PSFS, scale of 0-10)^d^	4.4 (2.5)	4.7 (2.6)	4.1 (2.4)
Physical function (PROMIS, T score)^d^	32.8 (3.9)	32.8 (4.0)	32.9 (3.9)
Aerobic activity (RAPA 1, scale of 1-7)^d^	3.7 (1.2)	3.8 (1.2)	3.5 (1.2)
Strength and flexibility (RAPA 2, scale of 0-3)^d^	1.4 (1.2)	1.5 (1.2)	1.3 (1.2)
Concomitant medications (take regularly, at least 5 d/wk)			
Acetaminophen	86 (23)	38 (21)	48 (26)
NSAIDs	107 (29)	54 (29)	53 (28)
Opioid	47 (13)	19 (10)	28 (15)
Gabapentin or pregabalin	117 (32)	55 (30)	62 (34)
Antidepressant	188 (51)	92 (51)	96 (52)
Antianxiety or sleeping medication	110 (30)	49 (27)	61 (32)
Muscle relaxants	84 (23)	43 (23)	41 (22)
Marijuana or cannabidiol	45 (12)	27 (15)	18 (10)
No prescription medications	51 (14)	26 (14)	25 (14)
Substance use (daily or almost daily in the last 12 mo [TAPS 1])			
Tobacco	54 (14)	26 (14)	28 (15)
Drinking 4 to ≥5 d/wk	57 (15)	28 (14)	29 (15)
Marijuana or cannabidiol	26 (7)	14 (7)	12 (6)
Prescription misuse	14 (4)	5 (3)	9 (5)

Abbreviations: BPI, Brief Pain Inventory; FIQR, Fibromyalgia Impact Questionnaire Revised; GAD 7, Generalized Anxiety Disorder 7; FIQR, Fibromyalgia Impact Questionnaire Revised; MAF; Multidimensional Assessment of Fatigue; MEP, movement-evoked pain; NRS, numeric rating scale; NSAIDs, nonsteroidal anti-inflammatory drugs; PCS, Pain Catastrophizing Scale; PHQ-8, Personal Health Questionnaire 8; PROMIS, Patient-Reported Outcomes Measurement Information System; PSD, Polysymptomatic Distress Scale; PT, physical therapy; PT-TENS, PT plus transcutaneous electrical nerve stimulation; SSS, Symptom Severity Scale; PSFS, Patient-Specific Functional Scale; RAPA, Rapid Assessment of Physical Activity; RUCA, Rural-Urban Commuting Area; TAPS, tobacco, alcohol, prescription medication, and other substance use; WPI, Widespread Pain Index.

^a^ Unless otherwise indicated.

^b^ Higher score indicates worse.

^c^ Baseline demographics for fibromyalgia-positive and fibromyalgia negative are in eTable 1 in Supplement 2.

^d^ Higher score indicates better.

### Primary Outcome

Movement-evoked pain on day 60 was significantly lower during TENS in the PT-TENS group when compared with the PT-only group in the randomized phase (group mean difference, −1.2; 95% CI, −1.6 to −0.7; *d* = 0.46). When missing data were imputed using multiple imputation models, findings were similar to the observed data (group mean difference, −1.1; 95% CI, −1.6 to −0.7) (eTable 3 in Supplement 2). Significant group differences were observed for both the fibromyalgia-positive (mean score, −1.0; 95% CI, −1.5 to −0.5) and fibromyalgia-negative (mean score, −1.6; 95% CI, −2.6 to −0.7) participants (eTable 4 in Supplement 2).

**Figure 2.**
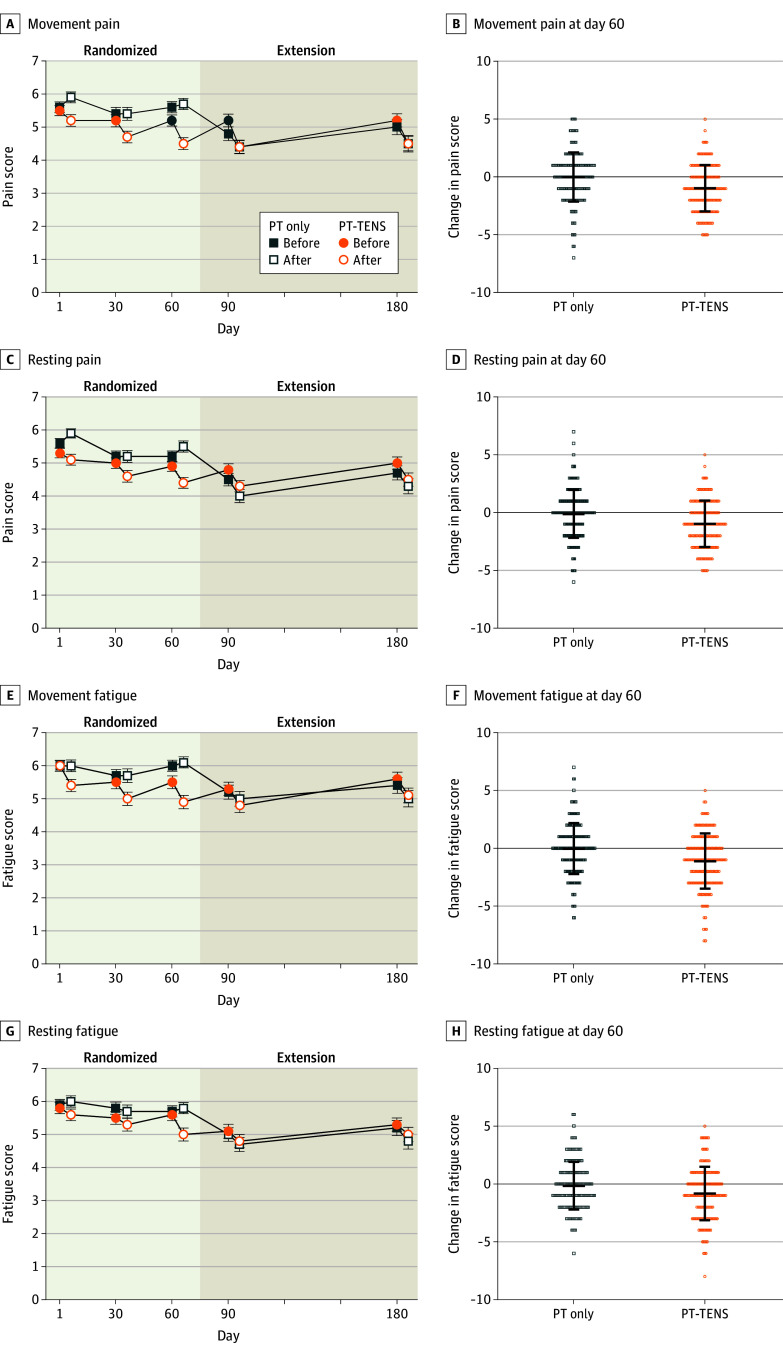
Changes in Movement-Evoked Pain, Resting Pain, Movement-Evoked Fatigue, and Resting Fatigue Before and During Transcutaneous Electrical Nerve Stimulation (TENS) Through Day 180 Line graphs show the mean (SEM) data before and after 30-minute application of TENS on each day while the TENS unit was on. The physical therapy (PT)–only group tested all measures before and after 30 minutes of rest on each day through day 60 (randomized phase) and before and after 30 minutes of TENS during the extension phase on days 90 and 180 while the TENS unit was on. The scatterplot shows individual-level data for the difference score on day 60 with means (SDs) for the physical therapy (PT)–only and PT-TENS groups. Data were measured on a scale of 0 to 10. For pain, 0 indicated no pain and 10 indicated worst pain imaginable. For fatigue, 0 indicated no fatigue and 10 indicated worst fatigue imaginable. Lower scores were observed in all measures after 30 minutes of TENS treatment for both pain and fatigue beginning on day 30 and persisting through day 180 for the PT-TENS group. There was no change in the measures for the PT-only group during the first 60 days. However, after receiving TENS, lower scores were observed in all measures after 30 minutes of TENS for the PT-only group on days 90 and 180 test while the TENS unit was on. Effect sizes were moderate for the difference in movement-evoked pain (*d* = 0.46), resting pain (*d* = 0.44), movement-evoked fatigue was (*d* = 0.48), and resting fatigue (*d* = 0.32). Error bars indicate SDs or SEMs.

**Figure 3.**
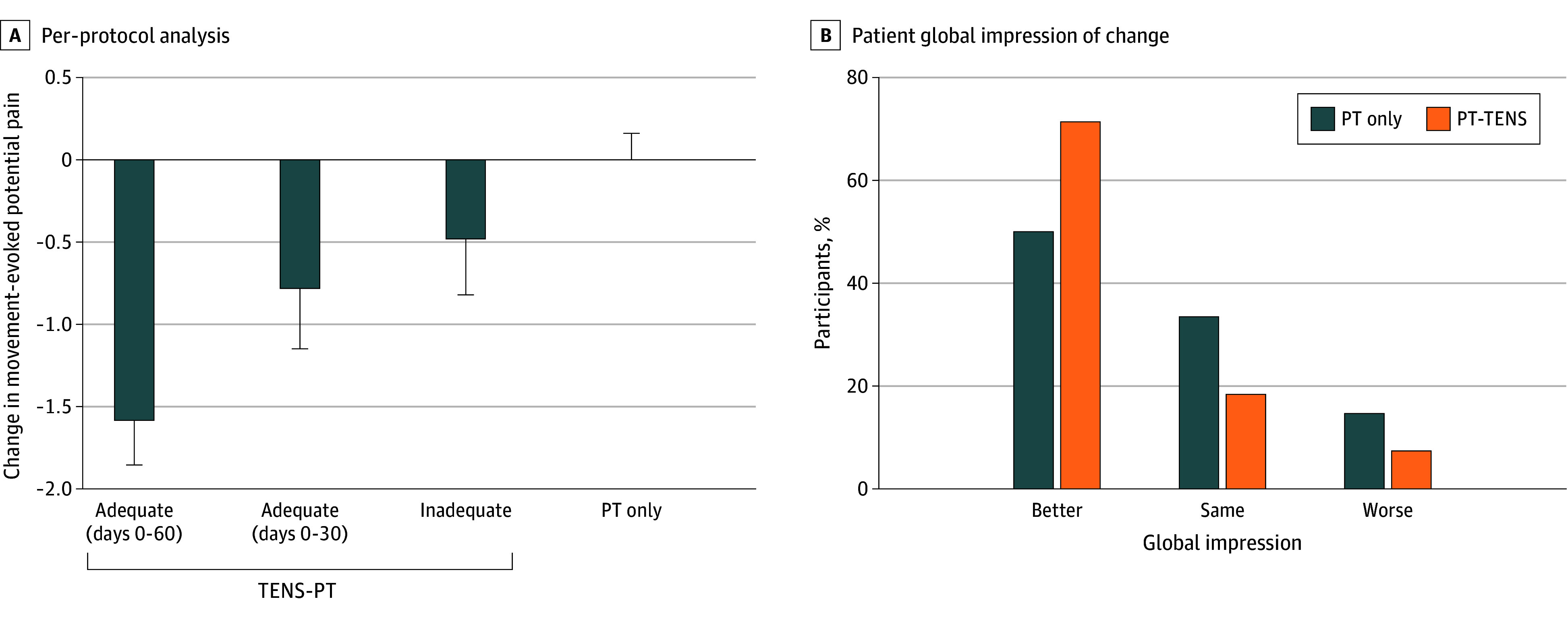
Movement-Evoked Pain and Patient Global Impression of Change Bar graph showing a dose-response effect in the per protocol analysis for change in movement-evoked pain from baseline to day 60 during the randomized phase. An adequate dose of transcutaneous electrical nerve stimulation (TENS) was defined as 8 times per month and 900 minutes per month. Movement-evoked pain decreased the most in those who used an adequate dose of TENS for the first 60 days (n = 76) followed by those who used an adequate dose for only the first 30 days (n = 30). Minimal changes in movement-evoked pain were observed for those who used less than an adequate dose of TENS throughout the 60-day period (n = 52). For comparison, the group who did not receive TENS (physical therapy [PT]–only group, n = 171) showed no change in pain. B. Bar graph showing results of the Patient Global Impression of Change on day 60 for the PT-only and PT-TENS groups. More respondents reported improvement in the PT-TENS group when compared with the PT-only group.

Movement-evoked pain was lower during TENS treatment by day 30 and remained lower through day 180 in the PT-TENS group using within-group comparisons (Figure 2A and Table 2; eTable 5 in Supplement 2). In the extension phase, movement-evoked pain was lower on days 90 and 180 during TENS treatment in the PT-only group compared with day 60 (Figure 2; eTable 5 in Supplement 2). A dose-dependent effect was observed with those who used TENS at the study-defined minimally effective dose through day 60 presenting the greatest decrease in movement-evoked pain followed by those who used the minimally effective dose for only the first 30 days (F_3,328_ = 9.05; *P* = .001) (Figure 3A; eFigure 1 and eTable 6 in Supplement 2).

**Table 2.** Primary and Secondary Outcomes Difference Scores in the Intention-to-Treat Analysis

Outcome	Baseline to day 60 difference, mean (95% CI)		PT-TENS vs PT only (day 60)	
	PT-TENS (n = 191)	PT-only (n = 193)	Group difference, mean (95% CI)	*P* value
Primary outcomes				
Movement-evoked pain (scale of 0-10)^a^	−1.0 (−1.4 to −0.7)	0.0 (−0.3 to 0.3)	−1.2 (−1.6 to −0.7)	<.001
Secondary outcomes				
Fibromyalgia impact (FIQR, scale of 0-100)^a^	−7.3 (−9.4 to −5.2)	−2.9 (−4.7 to −1.0)	−4.1 (−7.0 to −1.1)	.009
Resting pain (scale of 0-10)^a^	−1.0 (−1.3 to −0.7)	−0.1 (−0.4 to 0.2)	−1.0 (−1.4 to −0.5)	<.001
Pain severity (BPI, scale of 0-10)^a^	−0.4 (−0.7 to −0.2)	−0.3 (−0.5 to −0.0)	−0.3 (−0.6 to 0.0)	.070
Pain interference (BPI, scale of 0-10)^a^	−0.9 (−1.2 to −0.6)	−0.3 (−0.5 to −0.0)	−0.7 (−1.1 to −0.2)	.004
Movement-evoked fatigue (scale of 0-10)^a^	−1.1 (−1.5 to −0.7)	0.0 (−0.3 to 0.3)	−1.2 (−1.7 to −0.6)	<.001
Resting fatigue (scale of 0-10)^a^	−0.8 (−1.2 to −0.5)	−0.1 (−0.4 to 0.2)	−0.8 (−1.3 to −0.2)	.006
Global Fatigue Index (MAF, scale of 1-50)^a^	−3.2 (−4.6 to −1.8)	−0.9 (−2.1 to 0.2)	−1.9 (−3.8 to 0.0)	.05
Aerobic activity (RAPA 1, scale of 1-7)^b^	0.1 (−0.1 to 0.3)	0.2 (0.0 to 0.4)	0.1 (−0.2 to 0.4)	.48
Strength and flexibility (RAPA 2, scale of 0-3)^b^	0.3 (0.1 to 0.4)	0.4 (0.2 to 0.6)	−0.0 (−0.3 to 0.2)	.79
HEAL and exploratory outcomes				
Sleep (PROMIS, T score)^b^	0.2 (−0.5 to 0.9)	0.1 (−0.5 to 0.8)	0.3 (−0.6 to 1.1)	.51
Sleep duration (PROMIS), h^a^	0.1 (−0.1 to 0.3)	−0.2 (−0.4 to 0.1)	0.4 (0.1 to 0.7)	.03
Pain catastrophizing (PCS, scale of 0-52)^a^	−3.2 (−4.7 to −1.6)	−2.3 (−3.8 to −0.9)	−0.5 (−2.9 to 2.0)	.70
Depression (PHQ-8, scale of 0-24)^a^	−1.3 (−1.9 to −0.6)	0.0 (−0.6 to 0.7)	−1.3 (−2.2 to −0.3)	.01
Anxiety (GAD-7, scale of 0-21)^a^	−0.6 (−1.2 to 0.0)	−0.2 (−0.8 to 0.4)	−0.5 (−1.3 to 0.4)	.28
Activity average (PSFS, scale of 0-10)^b^	0.8 (0.3 to 1.2)	0.9 (0.4 to 1.4)	0.1 (−0.8 to 0.9)	.91
Physical function (PROMIS, T score)^b^	0.8 (0.3 to 1.3)	0.2 (−0.2 to 0.6)	0.6 (−0.1 to 1.2)	.09

Abbreviations: BPI, Brief Pain Inventory; FIQR, Fibromyalgia Impact Questionnaire Revised; GAD 7, Generalized Anxiety Disorder 7; HEAL, Helping to End Addiction Long-Term; MAF, Multidimensional Assessment of Fatigue; MEP, movement-evoked pain; NRS, numeric rating scale; PCS, Pain Catastrophizing Scale; PHQ-8, Personal Health Questionnaire 8; PROMIS, Patient-reported outcomes measurement information system; PSD, Polysymptomatic Distress Scale; PSFS, Patient-Specific Functional Scale; PT, physical therapy; PT-TENS, PT plus transcutaneous electrical nerve stimulation; RAPA, Rapid Assessment of Physical Activity; SSS, Symptom Severity Scale; WPI, Widespread Pain Index.

^a^ Higher score indicates worse.

^b^ Higher score indicates better.

### Secondary Outcomes

#### Pain

Resting pain was significantly lower during TENS on day 60 in the PT-TENS group when compared with the PT-only group (Figure 2B and Table 2). Compared with baseline, resting pain was lower during TENS treatment by day 30 and remained reduced through day 180. Pain interference was lower in the PT-TENS group (mean [SD] score, −0.9 [1.9]) compared with the PT-only group (mean [SD] score, −0.3 [1.6]; *P* = .004). A dose-response effect was observed for resting pain (eTable 5 in Supplement 1). Continued use of TENS for 6 months in the PT-TENS group resulted in significant improvements in multiple pain measures at day 180 (Table 2; eTable 5 in Supplement 2). After the PT-only group started using TENS, there were also significant improvements in multiple pain measures (Table 2; eTable 5 in Supplement 2).

#### Fatigue

Movement-evoked and resting fatigue on day 60 were significantly lower in the PT-TENS group compared with the PT-only group and remained lower through day 180 (Figure 2C-D and Table 2). In the PT-only group, movement-evoked and resting fatigue were lower during TENS treatment on days 90 and 180 (eTable 5 in Supplement 2; within-group comparison). There was an observed dose-response effect of TENS on movement-evoked and resting fatigue (eTable 6 in Supplement 2). Global fatigue (multidimensional assessment of fatigue) was also significantly lower in the PT-TENS group on day 60 and for both the PT-TENS and PT-only group on day 180 (Table 2; eTable 5 in Supplement 2).

#### Disease Impact

Disease impact (FIQR) was lower in the PT-TENS group on day 60 by a mean of −4.1 (95% CI, −7.0 to −1.1; *P* = .009) compared with the PT-only group and remained lower through day 180. Disease impact was also lower in the PT-only group during the extension phase (eTable 5 in Supplement 2).

#### Patient Global Impression of Change

On day 60, a greater number of participants in the PT-TENS group reported improvement (120 [72%]) compared with the PT-only group (86 [51%]) (χ^2^ = 16.7, *P* = .001) (Figure 3B). This effect remained for the PT-TENS group at day 180 (96 [67%]) and was similar to the PT-only group after using TENS in the extension phase (98 [77%]). Table 2 and eTable 5 in Supplement 1 detail the effects of TENS on additional psychological and functional measures.

### Responder Analysis

The PT-TENS group had more responders for movement-evoked pain (66 of 161 [41%]) compared with the PT-only group (22 of 169 [13%], χ^2^ = 32.0, *P* < .001). In addition, there were more responders for resting pain in the PT-TENS group (65 of 166 [41%]) compared with the PT-only group (37 of 172 [21%], χ^2^= 12.5, *P* = .004).

### Participant Experiences With TENS

On day 180 we asked participants about their experience with TENS. Of the 268 respondents, 217 (81%) said they found TENS helpful, 147 (55%) used TENS daily, and 66 (25%) used TENS at least once per week (eTable 7 in Supplement 2).

### Adverse Events

During the entire reporting period (6 months for PT-TENS and 4 months for PT only), there were no serious adverse events related to TENS. Minor adverse events included pain with TENS (27 [7.5%]), skin irritation with electrodes (24 [6.7%]), itchiness with TENS (22 [6.1%]), anxiety with TENS (15 [4.2%]), and nausea with TENS (4 [1.1%]) (eTable 8 in Supplement 2). Adverse events unrelated to TENS are given in eTable 9 in Supplement 2.

## Discussion

To our knowledge, this is the first trial of TENS for fibromyalgia in a clinical setting. The findings demonstrate effectiveness of this nonpharmacological intervention in reducing movement-evoked pain and suggest that the benefits of TENS are clinically meaningful in this population. Reductions in movement-evoked pain show a group difference of 1.2 of 10, with the clinically important change for movement-evoked pain reported as 1.1 (of 10).^25^ There were clinically significant improvements in other measures, including resting pain, pain interference, movement-evoked fatigue, resting fatigue, and fibromyalgia impact (FIQR), during the randomized phase. Similar reductions in pain and fatigue were observed in the PT-only group after starting TENS in the extension phase, typically after PT had been completed. The effectiveness of TENS occurred within 30 days and was sustained for 6 months. There was a dose-response effect for TENS with greater use associated with greater pain reductions. Forty-one percent of participants in the PT-TENS group achieved a 30% or greater reduction in movement-evoked pain, which is considered a clinically meaningful change for pain^25,26,27^ and moderately important by the consensus group Initiative on Methods, Measurement, and Pain Assessment in Clinical Trials (IMMPACT). Most individuals using TENS reported improvement (Patient Global Impression of Change). Lastly, most participants who completed the study found TENS helpful and still used TENS at least weekly. Thus, multiple lines of evidence support that TENS produces a clinically meaningful reduction in movement-evoked pain and other fibromyalgia symptoms, especially when provided at an adequate dose.

Clinical guidelines for fibromyalgia recommend treatment approaches specific to the individual, beginning with nonpharmacological therapies, particularly exercise, and progressing to pharmacological therapies, depending on patient response and disease severity.^10,28^ Systematic reviews^29,30,31^ show small-moderate effect sizes for aerobic exercise in reducing pain and fatigue (*d* = 0.31 for pain *and* 0.22 for fatigue; n = 27 randomized clinical trials) and moderate-large effects for function (*d* = 0.66-0.55). More recently, in individuals with fibromyalgia, lower disease impact (FIQR)^32,33^ was observed following tai chi when used clinically as a mind-body intervention, and lower pain interference^34^ was observed after transcutaneous direct current stimulation (*d* = 0.73) when combined with education and exercise. These studies^35,36,37^ support the use of activity-based interventions in treatment of fibromyalgia and suggest that combining interventions may improve effect sizes.

Due to the heterogenous nature of fibromyalgia, treatment often requires a multidisciplinary approach and is recommended in clinical guidelines.^38^ In the current study, most patients were already receiving pharmacological therapies and in the randomized phase received PT; thus, TENS was able to provide additional relief beyond that experienced with other interventions. We propose that TENS could be particularly useful as part of a multidisciplinary approach to provide a self-management tool that uniquely targets movement-evoked pain and fatigue, which are significant barriers to participation in exercise and daily activities.^18,19,20^

The current pragmatic cluster trial builds on FAST,^23^ with both trials showing a reduction in movement-evoked pain with TENS in individuals with fibromyalgia. Although FAST showed efficacy of TENS in reducing pain against placebo and no TENS under ideal conditions, FM-TIPS shows effectiveness in a clinical setting. When compared with the group not receiving TENS, the effect size for the change in pain in FM-TIPS (*d* = 0.46) was lower than that from FAST (*d* = 0.82). These differences in effect sizes could be due to less stringent exclusion criteria, addition of physical therapy to all groups, or greater comorbidities in the current study. Indeed, it is generally expected that pragmatic trials have lower effect sizes than explanatory trials primarily due to the less controlled nature of the trial, including greater heterogeneity in participants, clinics, interventions, and concomitant treatments^39^ and less control over adherence to the intervention.^40^ Importantly, FM-TIPS recruited individuals from diverse backgrounds across a range of socioeconomic and educational levels and both rural and urban environments and only excluded those with contraindications to, or prior use of, TENS. Thus, results from the current pragmatic trial are applicable to a wide range of individuals with fibromyalgia.

### Strengths and Limitations

The study’s strengths include a large and diverse sample recruited from 28 outpatient physical therapy clinics across 6 health care systems located in both rural and urban environments. Most of the more than 100 physical therapists who provided TENS and PT interventions had minimal to no experience with research yet were engaged in the study. The trial focused on movement-evoked pain, a substantial concern and barrier to participation in an effective exercise program and daily activities,^41,42,43^ which is not tested and targeted by currently available treatments. Together, these trial characteristics enhance the pragmatic nature of the trial and generalizability of the results.

The study also has limitations. We were unable to evaluate treatment effects on medication changes because electronic health records from PT clinics do not collect this information. Although we recruited individuals from racial and ethnic minority populations, these numbers were lower than the national average,^44,45^ which are likely related to the high recruitment we achieved of Midwest rural participants where populations of races other than White are lower (<5% Hispanic and <3% African American).^46^ Surprisingly, there was no significant effect of routine PT in individuals with fibromyalgia, which could be because only 69 of the 384 participants (18%) were referred to PT for fibromyalgia, and thus PT treatment may not have been directed toward fibromyalgia symptoms, limited insurance coverage for PT, adverse socioeconomic conditions, or poorer health.^47,48^

## Conclusions

The current study found clinically meaningful changes in pain, fatigue, and disease impact with TENS in individuals with fibromyalgia. TENS produced effects quickly, remained effective through 6 months, and continued to be helpful to most study participants. Importantly, the effect size observed in the clinical setting is similar to currently recommended pharmaceutical treatments and exercise, which were tested in more controlled conditions.^49,50,51,52,53,54,55^ Thus, TENS should be considered as a safe treatment option for fibromyalgia.

## Notes

Supplement 1.
Trial Protocol and Statistical Analysis Plan

Supplement 2. **eMethods.** Additional methods **eTable 1.** Demographic and outcome data for those meeting and not meeting the ACR Diagnostic Criteria **eTable 2.** Cluster-level consort information **eTable 3.** Results of different imputation methods on movement-evoked pain **eTable 4.** Sensitivity analysis for those who met the fibromyalgia diagnostic criteria (fibromyalgia-positive) and those who did not meet criteria at baseline (fibromyalgia-negative). **eTable 5:** Sustained effect of TENS use in fibromyalgia **eTable 6.** Per protocol analysis **eTable 7.** Participant experience with TENS **eTable 8.** Adverse events (AE) related to TENS **eTable 9.** Adverse events unrelated to TENS **eFigure.** Per protocol analysis
eReferences

Supplement 3.
Clinician Group Members

Supplement 4.
Data Sharing Statement
