# Errors in Tables 1 and 2

**DOI:** 10.1001/jamanetworkopen.2026.14239

**Published:** 2026-04-27

In the Original Investigation titled “Transcutaneous Electrical Nerve Stimulation and Pain With Movement in People With Fibromyalgia: A Cluster Randomized Clinical Trial,”^1^ published March 27, 2026, there were errors in Table 1 and Table 2. In Table 1, the Rapid Assessment of Physical Activity (RAPA) 2 scale for strength and flexibility should have been listed as 0 to 3. In Table 2, the second listing of “Fibromyalgia impact (FIQR, scale of 0-100)” should have instead been labeled as “Strength and flexibility (RAPA 2, scale of 0-3).” This article has been corrected.^1^
