# Supplementary material for: Transcutaneous Electrical Nerve Stimulation and Pain With Movement in People With Fibromyalgia: A Cluster Randomized Clinical Trial
Source: JAMA Netw Open. 2026 Mar 27;9(3):e262450. doi: 10.1001/jamanetworkopen.2026.2450 (PMC13032160; doi:10.1001/jamanetworkopen.2026.2450)
Supplement: Supplement 1. — Trial Protocol and Statistical Analysis Plan [file jamanetwopen-e262450-s001.pdf]

FIBROMYALGIA TENS IN PHYSICAL THERAPY (FM-TIPS): AN EMBEDDED PRAGMATIC CLINICAL TRIAL

Protocol Version: 11.0
Protocol Date: 12 August 2024

FM-TIPS

Principal Investigators
Kathleen A. Sluka, PhD, PT, FAPTA
University of Iowa Carver College of Medicine

Leslie J. Crofford, MD
Vanderbilt University Medical Center

Supported by:
National Institute of Arthritis and Musculoskeletal and Skin Diseases

The information contained herein is confidential and proprietary in nature and will not be disclosed to any third party without written approval of authorized designee. This document may be disclosed to the appropriate institutional review boards or to duly authorized representatives of the US Food and Drug Administration or a national regulatory authority under the condition that they maintain confidentiality.

TABLE OF CONTENTS

SIGNATURE PAGE 4
STATEMENT OF COMPLIANCE 5
LIST OF ACRONYMS, ABBREVIATIONS, AND DEFINITIONS OF TERMS 6
SYNOPSIS 7
1 STUDY OBJECTIVES 9
1.1 Primary Objective 9
1.2 Secondary Objectives 9
2 BACKGROUND 9
2.1 Rationale 9
2.2 Supporting Data 9
2.3 Risk/Benefit Assessment 11
3 STUDY DESIGN 11
3.1 Justification for Dose 12
3.2 End of Study Definition 12
4 SELECTION AND ENROLLMENT OF PARTICIPANTS 12
4.1 Inclusion Criteria 12
4.2 Exclusion Criteria 12
4.3 Study Enrollment Procedures 12
4.3.1 Participant Recruitment and Retention 13
4.3.2 Screening Documentation 14
4.3.3 Informed Consent 14
4.3.4 Randomization/Treatment Assignment 14
4.4 Participant Withdrawal Criteria 14
5 STUDY INTERVENTIONS/STUDY MEDICATION/STUDY DEVICE 14
5.1 Study Intervention, Administration, and Duration 14
5.2 Device Description 15
5.3 Handling of Study Intervention 15
5.4 Prohibited Concomitant Interventions 15
5.4.1 Prohibited Interventions 15
5.5 Participant compliance 15
6 CLINICAL EVALUATIONS/STUDY PROCEDURES 17
6.1 Schedule of Activities 17
6.2 Timing of Study Activities 19
6.2.1 First and Second PT Intake Visit 19
6.2.2 Day 1 Research Homework Before Next PT Visit 19
6.2.3 PT Treatment Visits (In-clinic) 20
6.2.4 30 Day Research Homework (Home +/- 5 days) 20
6.2.5 60 Day Research Homework (Home - 5/+10 days) 20
6.2.6 90 Day Research Homework (Home +/- 5 days) 21
6.2.7 180 Day Research Homework (Home +/- 5 days) 21
6.2.8 Study Intervention Discontinuation Evaluations/procedures 22
6.2.9 Final On-Study Evaluations 22
6.2.10 Protocol Deviations 22

6.2.11 Documentation of Fibromyalgia 22
6.2.12 Concomitant Medications/Treatments 22
6.2.13 Clinical Assessments 23
6.2.14 Questionnaires 23
6.2.15 Participant Adherence Assessments 24
7 MANAGEMENT OF ADVERSE EXPERIENCES 24
7.1 Adverse Event Reporting 24
7.2 Assessment and Recording of Adverse Events 25
8 MANAGEMENT OF UNANTICIPATED PROBLEMS 25
9 STATISTICAL CONSIDERATIONS 26
9.1 General Design Issues 26
9.2 Outcomes 26
9.2.1 Primary Outcome (including definition) 26
9.2.2 Secondary Outcome(s) 26
9.3 Sample Size and Accrual 27
9.4 Data Analyses 31
10 DATA COLLECTION 32
10.1 Data Management 32
10.1.1 Registration and Data Entry 32
10.1.2 Credentials 32
10.1.3 Password Strength 32
10.1.4 Two-factor Authentication 32
10.1.5 Data Backups & Retention 32
10.1.6 Logging 33
10.2 Data from Quell TENS Devices 33
10.2.1 Collecting Data from Quell TENS Devices 33
10.2.2 Data Transfer from NeuroMetrix 33
10.3 Quality Assurance 33
10.3.1 Remote Monitoring of Data 33
11 HUMAN SUBJECTS 34
11.1 Institutional Review Board (IRB) Review and Informed Consent 34
11.2 Participant Confidentiality 34
11.2.1 Certificate of Confidentiality 34
11.3 Study Modification/Discontinuation 34
12 PUBLICATION OF RESEARCH FINDINGS 34
13 REFERENCES 35
14 Appendix 1 BEsT TENS Precautions and Contraindications Decision Making 36

SIGNATURE PAGE

Study Number:

Principal Investigator Approval:

Signature: 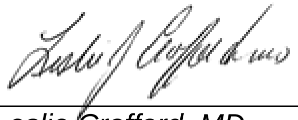 Date: 8/21/24

Name: *Leslie Crofford, MD*

Signature: 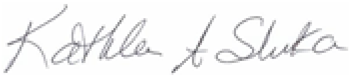 Date: 8/21/24

Name: *Kathleen Sluka, Pt, PhD*

STATEMENT OF COMPLIANCE

1. The trial will be carried out in accordance with International Conference on Harmonization Good Clinical Practice (ICH GCP) and the following:

    - United States (US) Code of Federal Regulations (CFR) applicable to clinical studies (45 CFR Part 46, 21 CFR Part 50, 21 CFR Part 56, 21 CFR Part 312, and/or 21 CFR Part 812)

    National Institutes of Health (NIH)-funded investigators and clinical trial site staff who are responsible for the conduct, management, or oversight of NIH-funded clinical trials have completed Human Subjects Protection and ICH GCP Training.

    The protocol, informed consent form(s), recruitment materials, and all participant materials will be submitted to the Central Institutional Review Board (cIRB) at the University of Iowa for review and approval. Approval of both the protocol and the consent form must be obtained before any participant is enrolled. Any amendment to the protocol will require review and approval by the cIRB before the changes are implemented to the study. In addition, all changes to the consent form will be cIRB-approved; a determination will be made regarding whether a new consent needs to be obtained from participants who provided consent, using a previously approved consent form.

LIST OF ACRONYMS, ABBREVIATIONS, AND DEFINITIONS OF TERMS

AE	Adverse Event
BPI	Brief Pain Inventory
CDE	Common Data Elements
CFR	Code of Federal Regulations
cIRB	Central Institutional Review Board
CRF	Case report form
CS	Clinically Significant
DCC	Data Coordination Center
DM	Data Management
DSMB	Data Safety Monitoring Board
eCRF	Electronic Case Report Form
EDC	Electronic data capture
EHR	Electronic Health Record
FDA	Food and Drug Administration
FIQR	Fibromyalgia Impact Questionnaire Revised
FM	Fibromyalgia
FM-TIPS	Fibromyalgia TENS in Physical Therapy Study
GAD7	Generalized Anxiety Disorder 7
GCP	Good Clinical Practice
HIPAA	Health Insurance Portability and Accountability Act
ICH	International Conference on Harmonization
IMM	Independent Medical Monitor
MAF	Multidimensional Assessment of Fatigue
MedDRA	Medical Dictionary for Regulatory Activities
NIAMS	National Institute of Arthritis and Musculoskeletal and Skin Diseases
NRS	Numerical Rating Scale
PCS	Pain Catastrophizing Scale
PEG	Pain, Enjoyment, General Activity Scale
PGIC	Patient Global Impression of Change
PHQ-8	Personal Health Questionnaire Depression Scale
PI	Principal Investigator
PROMIS	Patient-Reported Outcomes Measurement Information System
PSFS	Patient Specific Functional Scale
PT	Physical Therapy
RAPA	Rapid Assessment of Physical Activity
SAE	Serious adverse event
SOA	Schedule of Activities
TAPS	Tobacco, Alcohol, Prescription medication, and other Substance use
TENS	Transcutaneous Electrical Nerve Stimulation
SAS	Sit and Stand

SYNOPSIS

Study Title
Fibromyalgia TENS in Physical Therapy Study (FM-TIPS)

Objectives
Primary Objective: Determine if addition of Transcutaneous Electrical Nerve Stimulation (TENS) to routine physical therapy improves movement-evoked pain in patients with fibromyalgia (FM).

Secondary Objectives:

- Determine if addition of TENS to routine physical therapy improves disease activity and symptoms, increases adherence to physical therapy, increases the likelihood of meeting patient specific functional goals, and reduces medication use.
- Examine the feasibility of implementing TENS into routine PT care for FM using semi-structured exit interviews of patients and PTs.

Design and Outcomes
This study is a pragmatic clinical trial in outpatient PT practices utilizing cluster randomization by facility, and facility size. Sites will be randomized to enroll all eligible and consented participants with FM to TENS with PT, or PT only (no TENS). We are identifying 25-35 clinics across 6 healthcare systems in both rural and urban settings. We will enroll 450 patients that self-report a clinician diagnosis of FM. At each site, TENS (or no TENS) will be applied during each PT visit along with individualized PT treatment specified by the physical therapist. Participants randomized to TENS intervention sites will receive TENS units and electrodes at baseline to be applied to the cervical and lumbar regions. They will take the units home and bring the units back to their PT clinic visits with instructions for use at home while active and during their exercises. The physical therapist at each clinic will complete routine documentation of treatment. Variables including adherence to treatment will be extracted from each site's EHR. Additional patient- reported assessments will be obtained from the participants at home the day of their second PT visit and at 30, 60 (primary outcome), 90, and 180 days following enrollment.

Participants will be identified after they are referred for PT for treatment of FM or chronic pain (pain lasting more than three months). At their first (intake) physical therapy visit, they will be provided information about the study that can be accessed on paper or on-line. They will perform an electronic screening form on the first visit if interested in participating. Participants will provide eConsent after eligibility is confirmed at the initial PT visit. At the second PT visit after eConsent is signed, participants will be provided a link to access their survey queue within REDCap™ for data capture and accessing the case report forms. Participants enrolled at a TENS site will then be instructed in use of TENS and the TENS unit with electrodes will be provided. Those participants enrolled at a no TENS site will skip the instruction in use of TENS and TENS units will not be provided until after day 60 assessments are completed at which time they will be instructed in use of TENS by the study team remotely. All participants will be instructed to capture baseline assessments at home (called Research Homework) before their next PT visit. Pain and fatigue will be rated before and following their first TENS treatment (or no TENS) at home (day 1). At 30, 60, 90, and 180 days following enrollment, participants will complete all assessments at home, including pain ratings at rest and with movement along with questionnaires (see below for a list of questionnaires).

Interventions and Duration

For the TENS group, participants will be given TENS devices and electrodes following enrollment during their second PT visit. TENS will be applied to the upper and lower back with butterfly electrodes using the following parameters: mixed frequency (2-125Hz), strong but comfortable intensity, variable pulse duration from 100-250 microseconds. TENS will be applied at home when the patient is active and doing exercises for 2 hours per day and should be brought back to PT visits and used during PT treatment.

For the no TENS group, participants will be given TENS units after the primary endpoint is collected at 60 days. After completion of research homework for Day 60, TENS units will be mailed to the participants. The same instructions for use will be given to the participants as were given to the participants in the TENS group. TENS instructions will be done virtually with a study team member.

<u>Sample Size and Population</u>
Approximately 450 participants will be enrolled at 25-35 practice sites: half of the clinics will be randomized to TENS and the other half of the clinics will be randomized to no TENS.

<u>Inclusion Criteria:</u>
1. Age 18 or above
2. Clinician diagnosis of FM
3. Referred for land-based PT
4. Referred for treatment for FM or chronic pain (pain lasting more than 3 months)
5. Able to provide informed consent
6. Fluent in reading English
7. Willing to use TENS

<u>Exclusion Criteria:</u>
1. Contraindications to TENS use (See Appendix 1 for Precautions and Contraindication references):
    a. Pacemaker, defibrillator, implanted neurostimulator or implanted device
    b. Epilepsy
    c. Currently pregnant or plan to become pregnant in the next 6 months
    d. Allergic reaction to patches with gel
    e. Current treatment for cancer
2. Currently enrolled in another pain control study.
3. Use of TENS within the last 30 days.
4. Clinically unstable medical or psychiatric issues

1 STUDY OBJECTIVES

1.1 Primary Objective

The primary objective of the study is to determine if addition of TENS to routine PT improves movement-evoked pain in patients with fibromyalgia (FM).

1.2 Secondary Objectives

The secondary objectives of the study are to:

- Determine if addition of TENS to routine physical therapy improves disease activity and symptoms, increases adherence to physical therapy, increases the likelihood of meeting patient specific functional goals, and reduces medication use.
- Examine the feasibility of implementing TENS into routine PT care for FM using semi-structured exit interviews of patients and PTs.

2 BACKGROUND

2.1 Rationale

FM is a chronic pain condition characterized by widespread musculoskeletal pain, tenderness and stiffness associated with fatigue and sleep disturbance. A recent meta-analysis of 65 studies that included more than 3 million people world-wide showed the prevalence of FM is approximately 2 % overall and 4 % in women[1]. Pharmacological interventions are modestly effective for FM with most individuals continuing to experience activity-limiting pain and fatigue despite use of multiple drugs[2,3]. A recent population-based study reported that 22% of FM patients were using chronic opioids and 19 % were using chronic benzodiazepines[3]. It has become increasingly recognized that non-pharmacological interventions should be considered first-line treatments for chronic pain[4–6] and can be used as the initial treatment or added to pharmacological approaches. Despite clinical recommendations, non-pharmacological treatments are underutilized for chronic pain.

Inclusion criteria for the study include participants with a diagnosis of FM, or with a chronic pain condition (pain lasting more than three months). Individuals with FM have widespread pain affecting the trunk and extremities. FM is often a co-morbid condition that co-occurs with other chronic pain conditions, and those with FM have worse outcomes in PT. Treatment of FM has the potential to enhance outcomes for all conditions. Individuals with FM are not routinely referred to physical therapy but are referred for other types of chronic pain (back, neck, shoulder, knee). This criteria allows for identification of more individuals with FM, and is unlikely to affect baseline levels for the primary outcome measure of movement-evoked pain.

TENS is a non-pharmacological intervention that delivers electrical current through the skin for pain control. Prior work shows that TENS activates endogenous inhibitory mechanisms, including release of endogenous opioids in the central nervous system, to reduce central excitability[7,8]. Furthermore, clinical studies show TENS reduces postoperative opioid consumption.

2.2 Supporting Data

Our multi-institutional and multi-disciplinary research group recently completed an NIH/NIAMS funded(UM1 AR063381) randomized placebo-controlled clinical trial (N=301), FAST: Fibromyalgia Activity Study with TENS (NCT01888640), that showed Active-TENS (compared

to Placebo-TENS or No-TENS) reduced pain and fatigue during movement and at rest acutely (during the first application) and following 4 weeks of daily use. After the randomized phase, all groups received Active-TENS – there was sustained improvement in the Active-TENS group and equivalent improvement in the groups originally randomized to Placebo-TENS or No-TENS. Dramatic improvement in the global rating of change occurred for the Active-TENS group when compared to Placebo-TENS or No-TENS groups. TENS was well tolerated with <5% of participants reporting pain with TENS or irritation with the electrodes, which were the most common adverse events.

2.3 Risk/Benefit Assessment

The following are potential risks associated with the TENS unit:

1. Local skin irritation, caused by the adhesive in the TENS electrode pad, may occur.
2. Mild redness at the site of the TENS stimulation and a burning or pricking sensation if insufficient gel is used.
3. Discomfort or aggravation of pain from TENS.

Potential benefits of TENS include:

1. Reduced pain and fatigue at rest or with movement.
2. Improvement in symptoms of FM.
3. Improvement of functional status.
4. Reduced medication use.

An additional risk associated with the Sit and Stand Test is the potential for falls when completing this test at home.

There is a potential risk to personal information by connecting to a fraudulent or incorrect Quell app when study participants download the incorrect app for use with the TENS units. To prevent the download of an incorrect app to be used with the TENS units, instructions are given to study participants to only download the app during training sessions with study staff.

There is a possibility that the TENS unit could malfunction and increase the level of intensity on its own. If a potentially defective unit is identified by a participant with this issue, participants are advised to discontinue use of the unit and to alert the study team. The study team will send a prepaid box for the return of the unit and will replace the potentially defective unit with a new unit.

3 STUDY DESIGN

This study is a cluster-randomized, pragmatic trial conducted with approximately 450 participants across 25-35 physical therapy clinic sites from 6 health systems. We will employ a stratified randomization based on health system and size of the practice site so that half of the sites will provide TENS in addition to PT for each enrolled participant and the other half of the sites will provide routine individualized PT treatment. In the clinics randomized to standard of care, we will have specific instructions for them not to prescribe TENS to their FM patients. We recognize that some patients may be exposed to TENS through other means (relatives, friends) thus participants in the no TENS sites will also be instructed not to use TENS to prevent cross-ins; however, we believe our sample is sufficiently large to account for this potential exposure of TENS in a no TENS group. Furthermore, participants randomized to the no TENS group will receive TENS after the primary outcome data is collected at day 60 which may also prevent cross-ins and provide equipoise. At the completion of the study, we will specifically ask each participant about their TENS use, regardless of the randomization group. Primary analyses will be based on intention-to-treat.

Participants will be followed for a minimum of 60 days up to a maximum of 180 days with data collection from home at 1, 30, 60, 90, and 180 days following enrollment. The primary outcome variable will be assessed at day 60, which would be approximately at the completion of prescribed PT. Data collected from the PT clinics electronic health records (EHR) periodically

will include ICD 10 coding, the types of treatments (CPT codes), start and end dates for the episode of care, number of visits scheduled, and the number of visits attended. All patient reported measures will be obtained at home via computer, tablet, or phone.

3.1 Justification for Dose

TENS dose was determined on results of prior clinical trials. Most pertinent, the dosing for TENS used in the current study is the same as that provided in the Fibromyalgia Activity Study with TENS (FAST) study which showed a clinically significant effect reduction in pain and fatigue using mixed frequency TENS, during activity, with a strong, but comfortable intensity for two hours per day[10].

3.2 End of Study Definition

The primary endpoint of movement-evoked pain is assessed at Day 60. The end of the study will be the final Day 180 assessment or March 11, 2025, whichever comes first. All patient reported measures will be assessed at Day 180 for those who reach Day 180 prior to or on March 11, 2025, or at Day 60 or Day 90 for participants who will not reach Day 180 by March 11, 2025.

4 SELECTION AND ENROLLMENT OF PARTICIPANTS

4.1 Inclusion Criteria

Inclusion Criteria:

1. Age 18 or above.
2. Clinician diagnosis of FM
3. Referred for land-based PT
4. Referred for treatment for FM or chronic pain (pain lasting more than 3 months)
5. Able to provide informed consent.
6. Fluent in reading English
7. Willing to use TENS

4.2 Exclusion Criteria

Exclusion Criteria:

1. Contraindications to TENS use including:
    a. Pacemaker, defibrillator, implanted neurostimulator or implanted device
    b. Epilepsy
    c. Currently pregnant or plan to become pregnant in the next 6 months
    d. Allergic reaction to patches with gel
    e. Current treatment for cancer
2. Currently enrolled in another pain control study
3. Use of TENS within the last 30 days
4. Clinically unstable medical or psychiatric issues

4.3 Study Enrollment Procedures

Participants will be enrolled by the following procedure:

1. Participant identified by PT at initial visit
    - Information on study provided to the participant with a brochure, flyer, website

and contact number

- Screening questions for eligibility on Visit 1 by Tablet in REDCap™. Collect email/contact information.
- Electronic Informed Consent (eConsent) occurs after eligibility screen and before second PT visit.
- Once consent is obtained, REDCap™ sends automated email to the patient's PT clinic and study team.

2. Second PT Visit
    - PT verifies eConsent completion by email or REDCap™. If not complete, participant may decline or complete eConsent in clinic before study procedures begin.Provide instructions to participants for accessing surveys in REDCap™ on tablet, and accessing forms, and for TENS use and Quell smartphone app (if in TENS group).
    - TENS units serial numbers entered on a TENS accountability log (if in the TENS group).
    - REDCap™ link emailed to participants to complete Research Homework before PT Clinic Visit 3.
3. Third PT Visit
    - PT verities that baseline Research Homework (day 1) has been completed
    - If completed, proceed with PT using TENS (or no TENS) during treatment
    - If not completed, participant will be discontinued from the study and asked to return TENS units (if in the TENS group)

4.3.1 Participant Recruitment and Retention

Participants will be identified by the physical therapist at the first PT visit. Potential participants will perform an electronic screening on PT Visit 1. Contact information will be collected at screening on REDCap™. Eligible participants will be given information on the study and the potential participant will review the study materials in advance of their next PT visit. Contact information for the study team will be provided to answer any questions and address any concerns. The study team will contact eligible participants to follow up on eConsent. If the participant agrees to participate, they will sign the electronic consent.

Participants will continue PT with the therapist at their treatment location during their prescribed PT. The participant will complete patient-reported outcomes (Research Homework) at 1, 30, 60, 90, and 180 days electronically/paper if in the TENS group. Participants in the No TENS group will complete patient-reported outcomes (Research Homework) at 1, 30, 60, 65, 90 and 180 days. The study team will contact the participant with reminders when study activities are due.

Participants in the TENS group will be provided with all information including written instructions and electrodes for the TENS units during PT Visit 2. The no TENS group will be given TENS units, electrodes, and instructions after the primary endpoint at 60 days is collected to ensure equipoise and enhance retention of the no TENS group. A virtual follow up contact will be conducted by a study team member to instruct and review TENS use. A video providing visual instructions for TENS will be available to both groups on the study website.

4.3.2 Screening Documentation

Participants will be approached at their initial PT intake visit and complete the eligibility checklist on the tablet via REDCap™. If the participant meets eligibility, they will be provided information on the study to review prior to their next PT visit.

4.3.3 Informed Consent

Participants will be provided extensive information on the study in the form of links to web-based study information that includes videos about the study and about TENS. They will have the opportunity to review the consent document online. If requested, they will be provided a paper description of the study and consent document. The potential participant will have the opportunity to ask questions by directly contacting study personnel before their second PT visit or at their second PT visit.

4.3.4 Randomization/Treatment Assignment

Clinic sites will be randomized to provide TENS or no TENS along with individually prescribed PT treatments. All participants enrolled at a specific site will be in the same group: half the clinics will enroll participants into the TENS plus PT group (TENS Group) and the other half will enroll participants into the No TENS plus PT group for 60 days. Potential differences among the clinics will be taken into account by stratifying randomization so that clinics randomized to either TENS or no TENS will be as similar as possible.

4.4 Participant Withdrawal Criteria

Participants may be withdrawn from the trial for any number of reasons, including participant request or adverse events. If the patient does not complete their baseline assessment prior to their 3rd PT visit, they will be withdrawn from the study. If a participant wants to stop TENS use, they will be encouraged to remain in the study off study intervention and to complete the patient-reported outcomes assessments at the scheduled intervals. Study staff team members will contact the participant to determine the reason for withdrawal and complete the treatment termination CRF. There is no plan to replace participants who stop treatment. Ongoing contact including study newsletters will be used to minimize loss to follow-up and missing data. Providing TENS units to those participants randomized to no TENS following the day 60 primary endpoint is an additional strategy to minimize missing data and loss to follow-up.

5 STUDY INTERVENTIONS/STUDY MEDICATION/STUDY DEVICE

5.1 Study Intervention, Administration, and Duration

Participants in the TENS group will be given two TENS devices and electrodes on PT Visit 2. TENS will be applied to the upper and lower back with butterfly electrodes using the following parameters: mixed frequency (2-125Hz), strong but comfortable intensity, variable pulse duration from 100-250 microseconds. TENS will be applied during PT exercise treatment at the clinic and will be used at home when the patient is active or while doing exercise/activity for up to 2 hours per day.

The participants enrolled in the TENS group will use the device during the time of their PT (expected to be approximately 6 weeks but variable according to the individualized PT treatment plan), and after they complete PT for a total of 180 days.

Participants enrolled in the no TENS group will receive TENS units and electrodes after they

complete the primary outcome assessments at 60 days following enrollment to use for the next 120 days. All participants will be allowed to keep their TENS units after the study.

5.2 Device Description

Quell Wearable Pain Relief™ is patented non-invasive nerve stimulation technology that provides relief from chronic pain. It consists of a device and an electrode that attaches to the device. For the two TENS units, one electrode is placed on the upper back and one is placed on the lower back. We will request that these TENS units be used for approximately 2 hours per day with activity. The device is controlled by a single button on the device or a smartphone application (app). Quell provides pain relief by comfortably stimulating the nerves that carry normal, non-painful sensations to your brain, which decreases the perception of pain.

The Quell 2.0 is an FDA Class II Medical Device with FDA 510(k) clearance K152954. Indications for use: "Quell is intended for use as a transcutaneous electrical nerve stimulation device for the symptomatic relief and management of chronic intractable pain." The Quell TENS unit is a small device (98 mm x 74 mm x 11 mm) that will come with 2 charger cables and 2 charger adapters.

The FM-TIPS team will provide butterfly electrodes to the participants for the full 6 months of participation in the study (or 4-months for the no-TENS groups). These are commercially available electrodes with wires to connect the TENS unit to the electrodes.

5.3 Handling of Study Intervention

Sites will be provided with a TENS unit accountability log to complete. The log will document receipt of TENS units, serial number of each unit, and ID of the participant to whom the TENS unit was dispensed. TENS units not dispensed to participants will be returned to the study team.

5.4 Prohibited Concomitant Interventions

5.4.1 Prohibited Interventions

Participants enrolled in a clinic randomized to no TENS will be asked to not use TENS therapy during the first 60 days of study participation. After the 60-day collection of the primary endpoint, participants in no TENS clinics will be given TENS units with instructions on how to use the device.

Participants will not be required to change any of their prescribed medications during the study and no specific medications will be prohibited.

5.5 Participant compliance

The TENS unit collects data on usage electronically. Participants will be expected to connect device to a phone application once per week when their data will be uploaded to a cloud. We will receive regular information on all units utilized from Quell. Usage will be assessed in terms of number of 60-minute sessions and total number of minutes. In our previous study the total number of minutes was used for the per protocol analysis. As the current study will be based on intention to treat, usage information will also be used primarily for the per protocol analysis.

6 CLINICAL EVALUATIONS/STUDY PROCEDURES

6.1 Schedule of Activities

Evaluation	First and Second PT Intake Visit*	Research Homework before PT Visit 3 (Day 1[1])	Treatment Visits	30 +/- 5 days Home[1]	60 - 5/+10 days Home[1]	65 - 5/+15 days Home (No TENS Group Only)[4]	90 +/-5 days Home[1]	180 +/- 5 days Home[1]
Eligibility Screen- REDCap™	X							
PSFS		X		X	X		X	X
Informed Consent/ eConsent Review	X*							
Resting NRS pain/fatigue[2]		X		X	X	X	X	X
Sit and Stand Test with NRS pain and fatigue pre-treatment[3]		X		X	X	X	X	X
TENS applied (or not) x 30 minutes		X	X	X	X	X	X[3]	X[3]
Demographics		X						
2016 FM Criteria		X						
FIQR		X		X	X		X	X
MAF		X			X			X
BPI		X		X	X		X	X
PROMIS-Physical Function		X		X	X		X	X
PROMIS-Sleep		X		X	X		X	X
PCS		X		X	X		X	X
PHQ-8		X		X	X		X	X
GAD-7		X		X	X		X	X
RAPA		X			X			X
PGIC				X	X		X	X
TAPS1		X			X			X

Concomitant Medications		X						X
Sit and Stand Test with NRS pain and fatigue post TENS or no TENS treatment after 30 min of answering questionnaires		X		X	X	X	X[3]	X[3]
Adverse Events				X	X		X	X
Barriers to TENS								X

** Informational activities (e.g., video explaining study, description of TENS) will be delivered at home between PT visits. Consent will be obtained electronically before or as the initial activity of the second PT visit. After the second PT visit, participants will complete Research Homework. Day 1 is designated as the day the baseline study data is collected at home. Baseline data collection should be completed within 10 days of the second PT visit.*

[1]Assessment completed on computer or tablet, phone, or paper at home. If the participant does not have access to a computer, the data will be collected over the phone by the study team and directly entered into the database, or on paper/mailed or Faxed.

[2]Resting NSR pain/fatigue and Sit and Stand Test completed pre and post TENS treatment.

[3]All participants apply TENS

[4]Day 65 visit is only performed by the No Tens group

6.2 Timing of Study Activities

Recruitment will take place over 30 months. The study will consist of an initial in-clinic screening for eligibility and information on the study, including the study website and contact information for questions. Participants will sign an electronic consent before their second PT visit. At the second PT visit participants will be instructed on TENS use and completion of assessments in REDCap™. Baseline data will be collected at home by the participant logging into Redcap and completing assessments. PT in-clinic treatment visits will be done as individually prescribed by the physical therapist, and data collection conducted at home 1,30, 60, 90, and 180 days after study enrollment. For those participants without internet access, Research Homework may be done on paper forms that will be either collected at their PT visit or mailed to the study team (self-addressed, stamped envelopes will be provided).

6.2.1 First and Second PT Intake Visit

First Visit

- PT identifies possible participant
- Complete eligibility screen on REDCap™, if interested
- If eligible, PT instructs participant to complete electronic consent before PT Clinic Visit 2.
- Provide study information, refer to study website and provide contact for study team.
- When consent is complete REDCap™ sends email to PT and study team.

Second Visit

- PT verifies eConsent completion by email or REDCap™. If consent is not complete, participant may decline or may complete eConsent in clinic before study procedures begin.
- PT provides instructions on:
  - Logging into REDCap™ and accessing forms
  - The importance of the Sit and Stand test and Research Homework
  - TENS use (if applicable)
- Complete PT treatment with physical therapist.
- PT provides TENS units, electrodes, and written brochures to TENS group.
- PT logs TENS unit serial numbers.
- Redcap link emailed to participant to complete Research Homework before PT Clinic Visit 3.

6.2.2 Day 1 Research Homework Before PT Visit 3

- Log into REDCap™ (if participant does not have access to the internet, data collection may be completed on paper and mailed or faxed to study team, or completed by phone with a study team member)
- Pre-treatment resting NRS for pain and fatigue
- Pre-treatment Sit and Stand Test with NRS for pain and fatigue.
- TENS applied (or not) for at least 30 min while completing the assessments below on tablet (no TENS participants go directly to completing the assessments)
- Complete the following assessments on REDCap™:
  - Demographics
  - 2016 FM Criteria
  - PSFS
  - FIQR
  - MAF

- BPI
- PROMIS-Physical function
- PROMIS-Sleep + Sleep duration
- PCS
- PHQ-8
- GAD-7
- RAPA
- TAPS1
- Medications

- Post-treatment resting NRS for pain and fatigue
- Post-treatment Sit and Stand Test with NRS for pain and fatigue
- REDCap™ sends email to PT and study team when Research Homework is complete

6.2.3 PT Treatment Visits (In-clinic)

- Apply TENS during exercise treatment (TENS sites and NO TENS sites after day 65 if have in-clinic visit)

6.2.4 30 Day Research Homework (Home +/- 5 days)

- REDCap™ sends participant reminder email to complete Day 30 Research Homework
- Log into REDCap™ (or alternative method) to complete the following assessments
  - Adverse Events
  - Pre-treatment resting NRS for pain and fatigue
  - Pre-treatment Sit and Stand with NRS for pain and fatigue
- Apply TENS (or not)
  - PSFS
  - FIQR
  - BPI
  - PROMIS Physical Function
  - PROMIS-Sleep + Sleep duration
  - PCS
  - PHQ-8
  - GAD-7
  - PGIC
- Post-treatment resting NRS for pain and fatigue
- Post-treatment Sit and Stand test NRS for pain and fatigue
- REDCap™ sends email to study staff that Day 30 Research Homework is complete.

6.2.5 60 Day Research Homework (Home - 5/+10 days)

- REDCap™ sends participant reminder email to complete Day 60 Research Homework
- Log into REDCap™ to complete assessments:
  - Adverse Events
  - Pre-treatment resting NRS for pain and fatigue
  - Pre-treatment Sit and Stand Test with NRS for pain and fatigue
- Apply TENS (or not)
  - PSFS
  - FIQR
  - MAF
  - BPI

- PROMIS Physical Function
- PROMIS-Sleep + Sleep duration
- PCS
- PHQ-8
- GAD-7
- PGIC
- RAPA
- TAPS1

- Post-treatment resting NRS for pain and fatigue
- Post-treatment Sit and Stand Test with NRS for pain and fatigue
- REDCap™ sends email to study staff that Day 60 Research Homework is complete.

6.2.5.1 No TENS Group 65 Day Telehealth Visit by Study Team (Home -5/+15 days)

- Participants in no TENS clinics will be sent a TENS unit and electrodes with written instructions and links to video instructions for use through end of study.
- Study Team provides Telehealth instruction for TENS use, TENS app, and baseline Research Homework with TENS
- Participant completes Baseline Research Homework:
    - Log into REDCap™
    - Pre-treatment resting NRS for pain and fatigue
    - Pre-treatment Sit and Stand with NRS for pain and fatigue
- TENS applied for 1st full treatment X 30 minutes
    - Post-treatment resting NRS for pain and fatigue
    - Post-treatment Sit and Stand with NRS for pain and fatigue
- REDCap™ email sent to study staff when TENS instruction visit complete

6.2.6 90 Day Research Homework (Home +/- 5 days)

- REDCap™ sends participant reminder email to complete Day 90 Research Homework
- Log into REDCap™ to complete assessments:
    - Adverse Events
    - Pre-treatment resting NRS for pain and fatigue
    - Pre-treatment Sit and Stand Test with NRS for pain and fatigue
- TENS applied X 30 minutes
    - PSFS
    - FIQR
    - BPI
    - PROMIS Physical Function
    - PROMIS-Sleep + Sleep duration
    - PCS
    - PHQ-8
    - GAD-7
    - PGIC

- Post-treatment resting NRS for pain and fatigue
- Post-treatment Sit and Stand with NRS for pain and fatigue
- REDCap™ email sent to study staff after Day 90 Research Homework is complete

6.2.7 180 Day Research Homework (Home +/- 5 days)

- REDCap sends participant reminder email to complete Day 180 Research Homework
- Log into REDCap™ to complete assessments:
    - Adverse Events
    - Pre-treatment resting NRS for pain and fatigue
    - Pre-treatment Sit and Stand Test with NRS for pain and fatigue
- TENS applied X 30 minutes (all participants)
    - PSFS
    - FIQR
    - MAF
    - BPI
    - PROMIS Physical Function
    - PROMIS-Sleep + Sleep duration
    - PCS
    - PHQ-8
    - GAD-7
    - RAPA
    - PGIC
    - TAPS1
    - Concomitant Medications
    - Barriers to TENS

- Post-treatment resting NRS for pain and fatigue
- Post-treatment Sit and Stand Test with NRS for pain and fatigue
- REDCap™ email sent to study staff when Day 180 Research Homework is complete

6.2.8 Study Intervention Discontinuation Evaluations/procedures

Participants will be allowed to keep and continue to use the TENS devices following completion of the study, but electrodes will no longer be supplied. Individual participants may discontinue treatment at their discretion but will be encouraged to continue use through the end of the trial and even if they stop using TENS to complete all assessments as scheduled. If a participant develops a contraindication to TENS use during the study (such as pregnancy), they will be discontinued from treatment but not from the study. Adverse events will be collected at all routine assessments. The only reason we will not attempt to obtain all the assessment information is withdrawal of consent.

6.2.9 Final On-Study Evaluations

Final on-study evaluations are at the Day 60, 90 or 180-day assessment as described above.

6.2.10 Protocol Deviations

Protocol deviations are not being collected in this study.

6.2.11 Documentation of Fibromyalgia

FM diagnosis by criteria will be performed using the 2016 ACR criteria. Participants may be enrolled even if they do not meet criteria if they have been told by a clinician that they have FM. Criteria-based diagnosis will be used as a variable for statistical analysis. Physical therapists will not be asked to make the FM diagnosis themselves.

6.2.12 Concomitant Medications/Treatments

We will collect categories of concomitant medications used for pain, anxiety/depression, and sleep on a dedicated case report form. We will also collect information on substance use with the TAPS1 instrument at enrollment and at the end of the study.

6.2.13 Clinical Assessments

Patient-reported outcomes will be obtained, but no other clinical parameters will be measured. TENS-specific adverse events and serious adverse events will be recorded on the CRFs.

6.2.14 Questionnaires

The following questionnaires will be self-administered by participants:

- **Resting NRS for pain and fatigue**-11-point scale
- **Sit and Stand Test –** quantifies movement-evoked pain and fatigue.
- **2016 FM Criteria**- diagnostic criteria for fibromyalgia.
- **PSFS (Patient Specific Functional Scale) –** quantifies activity limitation and measures functional outcomes
- **FIQR (Fibromyalgia Impact Questionnaire Revised)**- 21 items used to evaluate function, overall impact, and symptoms in patients with fibromyalgia.
- **MAF (Multidimensional Assessment of Fatigue)**-16 items measuring fatigue according to four dimensions: degree and severity, distress that it causes, timing of fatigue, and impact on various activities of daily living.
- **BPI (Brief Pain Inventory)**- 11 items measuring pain severity and interference with daily activities.
- **PROMIS-Physical function –** assesses information about physical activities.
- **PROMIS-Sleep+Sleep duration**- 8 items + sleep duration assesses sleep quality and quantity
- **PCS (Pain Catastrophizing Scale)**- 13 items measuring pain magnification, rumination, and helplessness.
- **PHQ-8 (Personal Health Questionnaire Depression Scale)**- 8 items measuring depressive disorders.
- **GAD-7 (Generalized Anxiety Disorder)**- 7 items measuring severity of various signs of generalized anxiety disorder.
- **RAPA (Rapid Assessment of Physical Activity)**- 9 items evaluating current level of physical activity.
- **TAPS (Tobacco, Alcohol, Prescription Medication, and other Substance use)**- 4 item screening tool for substance abuse.

- **PGIC (Patient Global Impression of Change)**- one item evaluating all aspects of patients' health and assesses if there has been an improvement or decline in clinical status.

6.2.15 Participant Adherence Assessments

We will routinely analyze TENS usage electronically as provided by the device. If participants are not using the TENS as prescribed, we will inform the physical therapist and follow up with text, email, or phone calls with the participant to encourage usage.

7 MANAGEMENT OF ADVERSE EXPERIENCES

7.1 Adverse Event Reporting

An adverse event in this study will be defined as an unfavorable and unintended sign or symptom temporally associated with the use of the TENS unit, falls while performing the Sit and stand Test during Research Homework, or another unfavorable sign or symptom limited to problems associated with specific study activities, fibromyalgia symptoms, or hospitalizations/ED visits (see Question 2 below). Pain associated with physical therapy treatment may worsen during the study but will not be classified as an AE.

Adverse events will be queried and recorded on the Participant Experience CRF at each data collection (days 30, 60, 90, and 180) by asking participants the following questions:

"STUDY RELATED

Have you experienced any of the following problems since your last report? (check all that apply)

- ☐ Fell during sit and stand test
- ☐ Fell or injured yourself during physical therapy exercise at home
- ☐ None of the above

Have you had any of the following changes to your health since your last report?

- ☐ A pacemaker, implanted neurostimulator or any other implanted device
- ☐ A new diagnosis of epilepsy
- ☐ A new diagnosis of cancer
- ☐ Have become pregnant
- ☐ None of the above

Did you experience any of the following since your last report?

Hospitalization? ○ Yes ○ No

i. If yes, please explain: ______________________

ii. Was the hospitalization related to your TENS use? ○ Yes ○ No

Emergency room visit? ○ Yes ○ No

i. If yes, please explain: ______________________

ii. Was the emergency room visit related to your TENS use?

TENS RELATED

Have you experienced any problems using the TENS units or electrodes since your last report? ○ Yes ○ No

If yes, what type of problem did you experience? (check all that apply)

- ☐ Anxiety with TENS
- ☐ Itchiness with TENS
- ☐ Nausea with TENS
- ☐ Pain with TENS
- ☐ Skin irritation with electrodes
- ☐ Other TENS related problems

Please list other problems: ______________________________”

Adverse events that are reported to study staff outside of regularly scheduled homework where the study team is recommending discontinuing TENS use or where the participant discontinued TENS use on their own will be collected by the study team and reported in REDCap on the Participant Experience Review Form. For any serious adverse events reported outside of regularly scheduled homework, the information will be collected by study staff and forwarded to the medical safety monitor who will review the information for a determination regarding the safety of the subject continuing TENS use in the study. The monitor will complete the Participant Experience Review Form, which will be entered into REDCap.

Anticipated adverse events with TENS use include:

- Skin rash or irritation from electrodes
- Pain with TENS

7.2 Assessment and Recording of Adverse Events

The study team may contact the participant when an adverse event has been reported to collect further information on the AE, if needed. The team will determine if the adverse event is serious, unanticipated, related to TENS treatment, and if any action needs to be taken, for example, discontinue TENS use, or if further reporting to regulatory authorities is necessary.

Pregnancy:
Pregnancy itself is not regarded as an AE. However, TENS is not recommended for use during pregnancy and the participant will be withdrawn from treatment and the outcome of all pregnancies that occur during paternal or maternal exposure to study device (spontaneous miscarriage, elective termination, normal birth or congenital abnormality) must be followed up and documented even after the participant has been withdrawn from treatment.

8 MANAGEMENT OF UNANTICIPATED PROBLEMS

Unanticipated problems will be defined as unexpected adverse clinical symptoms related to TENS or electrode use, or to specific unexpected non-TENS adverse events as identified in Section 7.1. These will be reported on the adverse event CRF and will be reviewed at each

DSMB meeting. If unanticipated AE are uncovered that occur in >5% of enrolled participants, the consent form will be modified, and the AE will be specifically queried on the AE CRF going forward. Participants who have completed the study will be notified of any newly recognized TENS-related AE.

9 STATISTICAL CONSIDERATIONS

9.1 General Design Issues

This will be a cluster randomized pragmatic trial. A stratified randomization procedure will be used to randomize clinics to either no-TENS or TENS added to standard of care (routine physical therapy). Sites will be stratified by health system and size of practice site. Half of the clinics will be randomized to standard of care only and half of the clinics will be randomized to TENS plus standard of care.

We hypothesize that using TENS in a PT setting is feasible and that FM patients using TENS will have reduced movement-evoked pain and other symptoms and are more likely to reach their therapeutic goals and reduce medication use.

9.2 Outcomes

9.2.1 Primary Outcome (including definition)

Reduction of movement-evoked pain measured by 11-point NRS for pain during the Sit and Stand Test (stand up and sit down five times) from baseline to 60 days will be used as the primary outcome variable. The severity of pain will be measured on a 0 to 10 scale at each time where 0 is no-pain and 10 is worst pain imaginable. The baseline measurement will occur at home before the first instance of the patient applying the TENS device (or no TENS for control group patients). The primary outcome variable will be obtained after using the TENS unit for 30 minutes during the Sit and Stand Test with NRS for pain (movement-evoked pain) at the 60-day time point. All outcomes will be collected by the participant at home during Research Homework.

Movement-evoked pain will be operationalized by having the participant perform a Sit and Stand Test (sit and stand five times) and rating their pain with an NRS. Movement-evoked pain during 5TSTS was measured in our previous randomized clinical trial, the FAST study, and the change in this measure was used to calculate the sample size for the current study.

9.2.2 Secondary Outcome(s)

Change in the resting pain score from baseline (pre-TENS) to 60 days (post-TENS session) will be used as a secondary outcome variable. The severity of pain will be measured on a 0 to 10 scale at each time where 0 is no-pain and 10 is worst pain imaginable. Other secondary outcomes include change in the following domains:

- Pain interference (BPI)
- FM disease activity (FIQR)

- Resting fatigue (NRS)
- Movement-evoked fatigue (NRS)
- Multidimensional assessment of fatigue (MAF)
- Rapid assessment of physical activity (RAPA)
- Patient global impression of change (PGIC)

An exploratory outcome of patient-specific function using the patient-specific functional scale (PSFS) will be evaluated. We will also collect additional HEAL-specified measures (see study visit schedule) and medications.

9.3 Sample Size and Accrual

The required sample size was calculated to assess the effect of TENS+PT versus PT alone on the primary outcome of change from baseline to day 60 visit in pain after PT using two-independent samples t-test with inflation factor of [1+(m-1)*ICC] applied to account for average cluster size (m) and intra-cluster correlation (ICC). From the FAST study, there was a standard deviation of 2 units for change in movement-evoked pain during the five times sit to stand (5TSTS) Test. There was a greater decrease in pain with active-TENS compared to placebo-TENS of 1.3 (95% CI: 0.34, 2.2) for movement-evoked pain during 5TSTS. Compared to no-TENS, there was a greater decrease in pain with active-TENS of 1.8 (95% CI: 1.0, 2.8) for movement-evoked pain during 5TSTS. For this cluster randomized pragmatic trial comparing TENS+PT vs. PT alone, sample size was determined such that the statistical test at the 0.05 significance level will be able to detect a difference of at least 1.0 in mean change in pain with 80% power. Participants randomized to treatment arm will be clustered by clinic since clinics will be randomized for ease of implementation and thus, an estimate of the intra-cluster correlation (ICC) is needed for the sample size calculation. With no prior estimate of ICC, the required sample size per treatment arm (or participants per facility) for the desired detectable mean difference of 1.0, assuming SD=2.0, was calculated for combinations of ICC values and number of facilities per treatment arm; see Table 1 for ICC values for a sample size <600 participants per treatment arm. In a pilot study examining a non-pharmacological intervention for pain by our group we show an ICC of 0.01, DeBar and colleagues used an ICC of 0.002 for sample size calculation for a chronic pain population, and Adams et al. showed widely varying ICCs between data sets but the majority of patient-reported outcomes were below 0.095 ICC. Therefore, originally, we calculated the study sample size for ICC value between 0.12 and 0.14. We were conservatively estimating sample size of complete data from 456 participants total with 228 per treatment arm and 9 to 12 facilities per arm, which would allow an ICC of 0.12 to 0.14. The dropout rate in the previous randomized clinical trial from our group, in the FAST study, was 12% at 30 days follow-up. Since follow-up is 60 days in the proposed study, we expect a higher drop-out rate. The drop-out rate includes those patients who complete the informed consent form but do not complete the baseline assessment as a part of day 1 home activities, as well as those patients who start study and dropout prior to day 60. To account for up to 24% drop-out rate, a total of 600 participants (300 per arm) to be enrolled was originally planned. Table 4 shows the needed number of participants per facility (total participants within treatment arm), for specified ICCs throughout the range of 9 to 12 facilities per treatment arm (F), to detect a mean difference of at least 1 unit (NRS, 0-10) in pain at the 0.05 significance level with 80% power. Numbers in black show where we would be powered to detect a difference based on number of

facilities and different ICCs for n=300/arm while the tan numbers show ICCs for >300 per treatment arm.

For example, if the ICC is 0.14, to reach 80% power with 12 clinics in each arm, we need to have complete data from 19 patients per clinic, 228 patients in each arm: 456 patients total.

It should be noted that, we expect to enroll on average 25 patients per clinic for the scenario with 12 clinics per treatment arm. We expect some variability on the number of patients enrolled from each clinic.

Number of clinics will be increased in case the originally randomized clinics are not enrolling enough patients. In such scenario, the original overall sample size of 600 patients will be enrolled from more centers. Enrolling same number of subjects from more clinics gives higher statistical power compared to enrolling the same number of subjects from less number of clinics. Therefore, the scenario of adding more clinics would allow a higher drop-out rate from 600 enrolled patients. The randomization of new sites will be stratified based on health system and size of the practice site consistent with the original randomization. We will conduct an interim ICC recalculation after the first 200 participants have been enrolled (approximately 1/3 of the sample) for the purpose of determining ICC for the primary outcome measure. This analysis is to assess the primary outcome variable only for the purpose of calculating the ICC value and will not require unblinding of randomization. Therefore, it does not have an impact on the type I error rate and there is no change in our planned analysis on our statistical analysis plan. This analysis will only be used to determine the final number of subjects per site should be changed based on the ICC value. There are no stopping rules.

ICC	Table 1: ICC values by facilities (F) per treatment arm; subjects per site (total subjects)			
	F=9	F=10	F=11	F=12
0.050	11 (99)	9 (90)	8 (88)	7 (84)
0.100	23 (207)	16 (160)	13 (143)	11 (132)
0.110	30 (270)	20 (200)	15 (165)	12 (144)
0.120	43 (387)	25 (250)	17 (187)	14 (168)
0.125	56 (504)	28 (280)	19 (209)	14 (168)
0.130	--	34 (340)	21 (231)	16 (192)
0.135	--	41 (410)	24 (264)	17 (204)
0.140	--	53 (530)	27 (297)	19 (228)
0.145	--	--	32 (352)	21 (252)
0.150	--	--	39 (429)	23 (276)
0.155	--	--	51 (561)	26 (312)

The original sample size calculation conservatively assumed the ICC values to change between 0.12 and 0.14, subjects to be enrolled from 12 clinics in each group and approximately equal number of subjects to be enrolled from each clinic. During the conduct of the study, we recruited some clinics, and some other clinics were not actively enrolling. It was also observed that the number of patients enrolled per clinic was variable. Coefficient of variation (CV) is the ratio of standard deviation (sd) to the mean (CV = sd / mean), in terms of the enrollment per clinic. Due to variable enrollment numbers per clinic, we needed to account for variation in the number of enrolled participants per clinic by applying CV of subjects per clinic in the sample size calculation.

The number of active clinics at the time of the ICC recalculation was 28 where 26 clinics (13 in each treatment) had at least one participant with day 1 and day 60 assessments. The observed CV was 0.6.
We met with Drs. Liz Turner and Patrick Heagerty from the NIH Collaboratory Biostatistics Core in November 2022 to discuss our sample size recalculation plan. They had two recommendations: 1) recalculating the ICC value based on the adjusted model, consistent with the final analyses. 2) Calculating the ICC and the Jackknife estimate of standard error (SE) of ICC, and calculating the sample size using both the current estimate of ICC and ICC + 1SE as a conservative estimate.

When the interim ICC recalculation was conducted to determine the current ICC value based on the adjusted model, consistent with the SAP, with ~228 enrolled subjects, the calculated ICC value was 0.05 with the Jackknife estimate of the SE of ICC is 0.07. Taking into account the variable sample size per clinic (CV = 0.6) and using 13 clinics/group, sample size was estimated at 450 enrolled subjects to obtain a final number of 342 participants completing day 60 based on a 24% drop out rate. This sample size provides >90% power for ICC = 0.05, and 81%

power for ICC = 0.10 and 78% power for ICC of 0.12 (ICC + 1SE = 0.05 + 0.07). The study sample was updated as 450 assuming ICC value of 0.10. The sample size recalculation was presented to NIAMS and the DSMB. Both NIAMS and the DSMB recommended approval of the change in sample size.

9.4 Data Analyses

Descriptive statistics (means, medians, percentages, standard deviations, and interquartile ranges) will be computed for demographic and baseline variables for each of the treatment groups. The distributions of continuous variables will be evaluated for normality. If data are not normally distributed, appropriate transformation will be applied, or non-parametric methods will be used. Demographic and baseline variables will be compared between treatment groups using two independent samples t-test for continuous variables, Wilcoxon-rank sum test for ordinal as well as non-normally distributed continuous variables, and Pearson Chi-square test for categorical variables. Variables that are found to significantly differ between the groups may be used as possible covariates in the comparison of outcome measures between the treatment groups.

Intent-to-treat (ITT) analyses will be conducted that will include all participants that have been randomized.

The primary endpoint to assess efficacy of TENS+PT compared to PT alone is change in movement-evoked pain during the Sit and Stand Test from baseline to 60 days. This will be tested using linear mixed model analysis for repeated measures with treatment group, time, and treatment*time interaction as fixed effects. Random effects include facilities (within treatment), and participants (within facility, within treatment). In fitting the mixed model, appropriate covariance structures for longitudinal measures within participant will be considered. Our initial approach will be to assess compound symmetry and heterogeneous compound symmetry covariance structures. However, we will also explore different covariance structures and will not only be limited to these two options. These decisions will be finalized during the UG3 phase of the proposed study. The final covariance structure will be selected based on Akaike Information Criteria (AIC) and Schwarz's Bayesian Information Criteria (BIC). From the fitted model, differences in mean change between treatment groups will be assessed by test for treatment*time interaction effect. It is expected that stratified randomization will lessen the need for covariate-adjusted analyses. However, in the event that adjusted analyses are necessary, a secondary comparison of the primary endpoint between groups will be made by expanding the linear mixed model to include covariates. Potential covariates include age, race, ethnicity, TENS dose/intensity, opioid use, and use of FM (for example, anti-depressants, α2-delta ligands) medications. In addition, opioid use at baseline as a possible effect moderator of TENS will be examined by including an opioid*treatment*time interaction in the model. If found to be significant, then secondary analyses to test for TENS efficacy by opioid status will be performed with p-values adjusted using Bonferroni's method for multiple comparisons. Statistical significance for efficacy of TENS+PT vs. PT alone will be based on a two-tailed test at the 0.05 significance level with treatment effect summarized as mean difference with 95% confidence interval. Similar analyses will be performed for secondary outcome measures.

In the case of subject drop-out, reasons for subject drop-out will be recorded and compared between treatment groups. Subject characteristics and outcome measures collected prior to drop-out for those that drop-out will be compared to those that complete the study. In the presence of missing data, under the assumption of missing at random (MAR), linear mixed model analysis, which can handle incompletely observed subjects and uses a likelihood estimation method, will provide correct likelihoods and lead to valid estimates. However, since the data under analysis cannot distinguish if data is MAR or it is missing not at random (MNAR), sensitivity analysis will also be performed. Methods for sensitivity analysis such as marginal delta adjustment, conditional delta adjustment, reference-based controlled imputation, and other

pattern mixture models will be considered. If needed, variables will be transformed to satisfy approximate normality before imputation and retransformed back to original scale. To avoid imputation bias, all variables in the substantive analyses and, if feasible, other variables in the study database predictive of the missing values or influencing the cause of missing data will be included. Multiple imputation analyses using SAS PROC MI and SAS PROC MIANALYZE will be conducted. Any differences in results of the primary analysis and the sensitivity analyses, and possible reasons will be reported.

10 DATA COLLECTION

10.1 Data Management

10.1.1 Registration and Data Entry

Participants will enroll in the study and be assigned a participant ID by entering data into a tablet provided to each clinic. Patient level, clinician level, and clinic level data for the FM-TIPS study will be stored electronically in the REDCap™ platform at University of Iowa. Data Access Groups will be created to ensure that clinicians only have access to patient data within their own clinic.

The REDCap™ platform is managed by the Biomedical Informatics group in the Institute for Clinical and Translational Science at the University of Iowa. Each study team member will be granted access to REDCap™ through a secure login by the study PI, study manager or their proxy.

10.1.2 Credentials

Study team members log into REDCap™ using their University of Iowa HawkID and HawkID password. HawkID and HawkID passwords are never shared between individuals. External collaborators on the study are granted a HawkID and HawkID password at the request of the University of Iowa Study PI. HawkID and HawkID passwords follow the University of Iowa Password Policy. https://its.uiowa.edu/hawkid/password

10.1.3 Password Strength

University of Iowa HawkID passwords must be a minimum of nine (9) characters long, using a combination of alphabetic, numeric, and special characters and cannot contain consecutive, repeated characters (e.g., aaaaa11111) and cannot contain a string of characters that match previous passwords.

10.1.4 Two-factor Authentication

REDCap™ supports Two-factor authentication using DUO. Two-factor authentication requires the user to verify their identity through the Duo Mobile App or through a text/phone call to the user’s registered number.

10.1.5 Data Backups & Retention

In the University of Iowa REDCap™ data platform, primary data is stored in an HCIS Data Center. Data backups are secured at a University of Iowa offsite Data Center. Physical system security includes two factor authentication. Operating system security includes: secure logins, firewalls, data encryption at rest, remote system logging and configuration and change management. Data backups are encrypted both in flight and at rest. Copies of data are

replicated to the remote data center every 15 minutes. There are 100+ point in time copies of data available at any time. Disaster recovery is tested annually.

10.1.6 Logging

REDCap™ automatically logs all user access to data on the system and project level, including page views.

10.2 Data from Quell TENS Devices

10.2.1 Collecting Data from Quell TENS Devices

Each study subject will be using two Quell devices, one designated as lower back and one designated as upper back. Designation of “Upper” and “Lower” is indicated on each unit above the Quell device serial number and is confirmed upon pairing the Quell device via Bluetooth to a smartphone application. The smartphone application will connect to Quell Health Cloud (QHC) via an account creation step, where participants enter their unique study ID (subject XXXX from site YY) into the email field as TIPSYYXXXX@quellrelief.com. This account creation step allows for all data on TENS usage from both devices to sync to a single Quell Health Cloud account identified by the account login credentials without personal identifiable information. The Quell TENS data (total number of minutes used per week and intensity) will be stored separately for each device by its designation/serial number. The data is stored in a segregated, secured location on NeuroMetrix servers.

10.2.2 Data Transfer from NeuroMetrix

Data collected by NeuroMetrix for the Quell TENS devices will be provided to the University of Iowa Clinical Trials Statistical Data Management Center on a regular basis.

10.3 Quality Assurance

The Data and Safety Monitoring Board (DSMB) is responsible for monitoring participant safety and study quality. A DSMB will be established by NIAMS through KAI. We recommend that the DSMB be comprised of a rheumatologist familiar with clinical trials in FM subjects and a physical therapist familiar with the use of TENS for pain, and clinical researchers familiar with studies using non-pharmacological management strategies in patient populations. TENS is an FDA-approved treatment for pain that has minimal risks. The DSMB will convene biannually or as decided by NIAMS.

The DSMB will review the protocol with emphasis on data integrity and patient safety issues, monitoring adverse events, protecting the confidentiality of the data and monitoring results, and making recommendations to NIAMS and Principal Investigator to continue or conclude the study.

10.3.1 Remote Monitoring of Data

Reports will be run to look for data discrepancies, missing or incomplete data. Participants may be contacted by study team members to inquire and resolve data discrepancies and/or missing data.

11 HUMAN SUBJECTS

Documented approval from the cIRB will be obtained for all participating clinics prior to clinical trial start, according to ICH GCP, local laws, regulations and organization. When necessary, an extension, amendment or renewal of the CIRB approval must be obtained.

Evidence of training in responsible conduct of research shall be on file for each study staff member as well as physical therapists at each practice site.

11.1 Institutional Review Board (IRB) Review and Informed Consent

This protocol and the informed consent document and any subsequent modifications will be reviewed and approved by the cIRB responsible for oversight of the study. A signed electronic consent form, approved by the cIRB, will be obtained from the participant.

11.2 Participant Confidentiality

All evaluation forms, reports, video recordings, and other records that leave the clinical study site will be identified only by the study specific Participant Identification Number (PID) to maintain participant confidentiality. All records will be kept in a secure location. All computer entry and networking programs will be done using study specific SIDs only. Clinical information will not be released without written permission of the participant, except as necessary for monitoring by CIRB, the NIH, and the OHRP.

11.2.1 Certificate of Confidentiality

To further protect the privacy of study participants, a Certificate of Confidentiality will be issued by the National Institutes of Health (NIH). This certificate protects identifiable research information from forced disclosure. It allows the investigator and others who have access to research records to refuse to disclose identifying information on research participation in any civil, criminal, administrative, legislative, or other proceeding, whether at the federal, state, or local level. By protecting researchers and institutions from being compelled to disclose information that would identify research participants, Certificates of Confidentiality help achieve the research objectives and promote participation in studies by helping assure confidentiality and privacy to participants.

11.3 Study Modification/Discontinuation

The study may be modified or discontinued at any time by the cIRB, the sponsor, the OHRP, the FDA, or other government agencies as part of their duties to ensure that research participants are protected. If the study is terminated or suspended, the study team will promptly inform study participants, and the IRB, and provide the reason(s) for the termination or temporary suspension.

12 PUBLICATION OF RESEARCH FINDINGS

Data from the research study will be published in peer-reviewed journals. The executive committee will develop a publication policy for use of data and authorship.

13 REFERENCES

1. Heidari F, Afshari M, Moosazadeh M. Prevalence of fibromyalgia in general population and patients, a systematic review and meta-analysis. Rheumatol Int 2017; 37:1527-39.
2. Okifuji A, Hare BD. Management of fibromyalgia syndrome: review of evidence. Pain Ther 2013; 2:87-104.
3. Vincent A, Whipple MO, McAllister SJ, Aleman KM, St Sauver JL. A cross-sectional assessment of the prevalence of multiple chronic conditions and medication use in a sample of community-dwelling adults with fibromyalgia in Olmsted County, Minnesota. BMJ Open 015;5: e006681.
4. Qaseem A, Wilt TJ, McLean RM, Forciea MA, Clinical Guidelines Committee of the American College of P. Noninvasive Treatments for Acute, Subacute, and Chronic Low Back Pain: A Clinical Practice Guideline from the American College of Physicians. Ann Intern Med 2017; 166:514-30.
5. Macfarlane GJ, Kronisch C, Atzeni F, et al. EULAR recommendations for management of fibromyalgia. Ann Rheum Dis 2017;76: e54.
6. Dowell D, Haegerich TM, Chou R. CDC guideline for prescribing opioids for chronic pain-United States. MMWR Recomm Rep2016:1-49.
7. Vance CG, Dailey DL, Rakel BA, Sluka KA. Using TENS for pain control: the state of the evidence. Pain Manag 2014; 4:197-209.
8. Sluka KA, Walsh DM. Transcutaneous Electrical Nerve Stimulation and Interferential Therapy. In: Sluka KA, ed. Pain Mechanisms and Management for the Physical Therapist. 2nd Edition ed. Philadelphia: Wolters Kluwer; 2016:203-23.
9. Bjordal JM, Johnson MI, Ljunggreen AE. Transcutaneous electrical nerve stimulation (TENS) can reduce postoperative analgesic consumption. A meta-analysis with assessment of optimal treatment parameters for postoperative pain. Eur J Pain 2003; 7:181-8.
10. Dailey DL, Vance CG, Rakel BA, Zimmerman MB, Embree J, Merriwether EN, Geasland KM, Chimenti R, Williams J, Golchha M, Crofford LJ, Sluka KA. A Randomized Controlled Trial of TENS for Movement-Evoked Pain in Women with Fibromyalgia. Arthritis & rheumatology (Hoboken, NJ). 2019 Nov 18 doi: 10.1002/art.41170. [Epub ahead of print]

Appendix 1

BEsT TENS Precautions and Contraindications Decision Making

CONTRAINDICATIONS https://www.ncbi.nlm.nih.gov/pmc/articles/PMC3031347/

Contraindication	Rationale	Evidence
**Implanted Devices**		
Pacemaker, Cardioverter defibrillator, Neuro-stimulators (brain or SC), Bone growth stimulators, Indwelling BP monitors	• TENS has been shown to interfere with these devices and should be avoided over or close to these areas<br>• For use with pacemakers, discuss the situation with a cardiologist and perform an initial TENS trial with Holter/ECG monitoring	MODERATE
**Body Regions**		
Carotid sinus	• Stimulation of baroreceptors may cause a drop in HR/BP<br>• Stimulation of the vagus and phrenic nerves may cause a laryngeal spasm.	LOW
Damaged skin	• Decreased skin impedance results in high current flow that may cause additional pain and tissue damage	STRONG
Reproductive organs, genitalia	• The risk of TENS for gametogenesis are unknown.<br>• Stimulation of reproductive organs or genitalia requires special training	ABSENT
Transcranial application	• Risks are not fully understood<br>• Risk of seizure<br>• Only therapists with advanced skills should apply transcranially	MODERATE
Eyes, oral cavity	• Effects of currents unknown<br>• No know indication	ABSENT
**Conditions**		
Cancer- local site<br><br>Distant to local site, see precautions	• The effect of TENS on cancer cells/metastasis is unknown<br><br>• Abnormal growths should be regarded as malignant until a diagnosis is rendered<br>• Patients presenting with both undiagnosed pain and a history of cancer in the last 5 years should not receive TENS<br><br>• TENS may be used for patients in palliative care when benefits of pain reduction outweigh risks<br>• Application of TENS at sites distant to the site of cancer is a precaution and not contraindicated.	LOW

Pregnancy-torso	• The effects of electrical current passage through the uterus have not been determined<br>• The greatest risk is during the first trimester<br>• Stimulation over the abdomen may lead to unwanted uterine contraction<br>• Endogenous opiates are stimulators of myometrial Contractions<br>• TENS is effective for labor pain	MODERATE
Epilepsy (head, neck and shoulder regions)	• TENS may induce seizures.	MODERATE
Thrombosis/Thrombophlebitis	• TENS may increase circulation increasing risk of dislodging a thrombus.	MODERATE
Hemorrhage	• Increase bleeding may occur in patients with current or recent hemorrhage or hemorrhagic condition	MODERATE
Recently radiated tissues	• Tissue may respond atypically due to radiation induced inflammation or scar tissue.<br>• Possibility of remaining malignant cells	MODERATE
Infection, Osteomyelitis, Tuberculosis	• May result in the spread of compartmentalized infections	LOW

PRECAUTIONS https://www.ncbi.nlm.nih.gov/pmc/articles/PMC3031347/

Precaution	Rationale	Evidence
**Pain**		
Undiagnosed pain	• Postponing proper treatment and risk of worsening of underlying condition	N/A
**Conditions**		
Cancer- distant to local site (Local site- see contraindications)	• Application of TENS at sites distant to the site of cancer is a precaution and not contraindicated.<br>• TENS may be used for patients in palliative care when benefits of pain reduction outweigh risks.<br>• Patients 5 years in remission are removed from the contraindication/precaution list.	LOW
Pregnancy-over acupuncture points at the knee, hand, ankle	• TENS over acupuncture points has been demonstrated to increase uterine contraction	MODERATE
Cognitive Impairment	• A patient needs to be able to perceive a safe intensity.<br>• Consider referral to physical therapy and/or training a caregiver in appropriate use	MODERATE
Cardiovascular disease	• Do not apply to chest<br>• Use at sites other than the chest wall	MODERATE
**Impairments/Body Regions**		
Extreme edema	• May adversely affect conduction of current to deeper tissues	N/A

Extreme adipose	• May adversely affect conduction of current to deeper tissues	N/A
Previous adverse TENS experience	• Refer to physical therapy to further evaluate	N/A
Visual impairment	• If using independently will not be able to determine unit settings	N/A
Trans thoracic applications	• Application of current across the chest (heart) is not necessary for pain relief<br>• Alternative electrode placements should be sought.	N/A
Impaired sensation, scar tissue	• Patients may be unable to provide feedback to properly set TENS intensity<br>• Consider referral to physical therapy	MODERATE

Level of Research Evidence

Strong	Clinical reports are consistent and suggest a potential for adverse reactions should TENS be used in the presence of this condition or on this body area. These clinical reports are supported by experimental evidence and/or by a strong biophysical rationale for the adverse reaction.
Moderate	The potential harmful effect has been demonstrated in experimental research using appropriate cell culture or animal models or when applied to health human subject; however, clinical evidence is either lacking or conflicting.
Low	There is a sound biophysical rationale to explain how TENS might cause an adverse reaction; however, there is no research evidence, either animal or clinical, to substantiate this response, or the evidence is contradictory.
Absent	No research, either experimental or clinical, has been found, and there is no known biophysical rationale to explain how the adverse reaction may occur.
N/A	These obvious precautions are included by the BEsT team. They are not documented in the attached references.

References

Electrophysical Agents- Contraindications and Precautions: An evidenced based Approach to Clinical Decision Making in Physical Therapy. Physiother Can. 2010;Fall62(5)1-80

Bellew JW, MIchlovits SL, Nolan, TP. Modalities for Therapeutic Intervention, 6th edition. FA Davis. Philadelphia, PA.2016. Chapter 11.

.

Docusign Envelope ID: 15F39A01-5329-4125-A15A-B19595C72975

Fibromyalgia TENS in Physical Therapy Study (FM-TIPS) Statistical Analysis Plan

A Cluster Randomized Study by Facility and Health Systems, Double Arm (TENS with PT versus PT only), Pragmatic Clinical Trial, and Feasibility of Implementation of TENS in PT Care in Participants with Fibromyalgia (FM)

Principal Investigators

Kathleen A. Sluka, PhD, PT, FAPTA
University of Iowa Carver College of Medicine

Leslie J. Crofford, MD
Vanderbilt University Medical Center

Emine Bayman, PhD
University of Iowa
Principal Investigator of Biostatistics Coordinating Center

Supported by:
National Institute of Arthritis and Musculoskeletal and Skin Diseases

VERSION 2.0
May 25, 2025

STATISTICAL ANALYSIS PLAN SIGNATURE PAGE

FM-TIPS: A Cluster Randomized Study by Facility and Health Systems, double arm (TENS with PT versus PT only), Pragmatic Clinical Trial, and Feasibility of Implementation of TENS in PT Care in Participants with Fibromyalgia (FM)

Principal Investigator Approval

DocuSigned by: Kathleen Sluka
BB2FE11A5F1044B...

Signature: ____________ **Date:** 6/13/2025
Name: Kathleen A. Sluka, PhD, PT, FAPTA
Affiliation: University of Iowa Carver College of Medicine

DocuSigned by: Leslie Crofford
738227482C4945E...

Signature: ____________ **Date:** 6/9/2025
Name: Leslie Crofford, MD
Affiliation: Vanderbilt University Medical Center

Biostatistics Coordinating Center Approval

DocuSigned by: Emine Ozgur Bayman
E2A88D4C5C4C488...

Signature: ____________ **Date:** 6/9/2025
Name: Emine Bayman, PhD
Affiliation: University of Iowa

Docusign Envelope ID: 15F39A01-5329-4125-A15A-B19595C72975

LIST OF ACRONYMS, ABBREVIATIONS, AND DEFINITIONS OF TERMS

AE	Adverse Event
BPI	Brief Pain Inventory
CDE	Common Data Elements
CFR	Code of Federal Regulations
cIRB	Central Institutional Review Board
CRF	Case report form
CS	Clinically Significant
DCC	Data Coordination Center
DM	Data Management
DSMB	Data Safety Monitoring Board
eCRF	Electronic Case Report Form
EDC	Electronic data capture
EHR	Electronic Health Record
FDA	Food and Drug Administration
FIQR	Fibromyalgia Impact Questionnaire Revised
FM	Fibromyalgia
GAD7	Generalized Anxiety Disorder 7
GCP	Good Clinical Practice
HIPAA	Health Insurance Portability and Accountability Act
ICH	International Conference on Harmonization
IMM	Independent Medical Monitor
ITT	Intention-to-treat
MAF	Multidimensional Assessment of Fatigue
MedDRA	Medical Dictionary for Regulatory Activities
MEP	Movement Evoked Pain
mITT	Modified intention-to-treat
NIAMS	National Institute of Arthritis and Musculoskeletal and Skin Diseases
PCS	Pain Catastrophizing Scale
PEG	Pain, Enjoyment, General Activity Scale
PGIC	Patient Global Impression of Change
PHQ-8	Personal Health Questionnaire Depression Scale
PI	Principal Investigator
PROMIS	Patient-Reported Outcomes Measurement Information System
PT	Physical Therapy
RAPA	Rapid Assessment of Physical Activity
SAE	Serious Adverse Event
SOA	Schedule of Activities
TAPS	Tobacco, Alcohol, Prescription Medication, and Other Substance Use
TENS	Transcutaneous Electrical Nerve Stimulation
5TSTS	Five Times Sit and Stand

Docusign Envelope ID: 15F39A01-5329-4125-A15A-B19595C72975

PREFACE

This Statistical Analysis Plan (SAP) describes the planned analyses for the FM-TIPS study, funded by the National Institutes of Health (NIH). The planned analyses identified in this SAP are intended to support the completion of a final report that will be prepared and presented to the study sponsor. All final, planned analyses identified in this SAP will be performed only after the last randomized study participant has completed the study, and all data have been cleaned and verified in accordance with applicable standard operating procedures at the FM-TIPS Data Coordinating Center (DCC). Once all data have been cleaned and verified, a "locked" version of the data will be used for reporting the final study results. Key statistics and study results will be presented verbally to the PI, DCC, Data and Safety Monitoring Board (DSMB), and Steering Committee (SC) for review and discussion following database lock and prior to completion of the final report.

The hypothesis test for the primary outcome measure to address the primary aim will be assessed at the 0.05 significance level. The same significance level will be used for all other secondary hypothesis tests on the secondary measures with no attempt to make any type of adjustment for multiplicity, unless otherwise specified.

1. STUDY DESIGN

1.1 Design

This study is a cluster-randomized pragmatic trial conducted where the initial proposed sample size was 600 across 24 physical therapy clinics. The original sample size was reduced from 600 based on a planned interim sample size recalculation following enrollment of the first ~200 participants and based on the planned interim assessment of intra-class correlation coefficient (ICC). A stratified randomization of practice sites will be employed based on healthcare system and size of the practice site (clinics) so that half of the clinics will provide transcutaneous electrical nerve stimulation (TENS) in addition to physical therapy (PT) (TENS+PT) for each enrolled participant and the other half of the clinics will provide standard of care routine individualized PT treatment (PT only). To avoid potential exposure to TENS in the clinics randomized to PT only, the clinics will be specifically instructed not to prescribe TENS to their fibromyalgia (FM) patients. Likewise, study participants in the PT only site that may be exposed to TENS through other means (relatives, friends) will also be instructed not to use TENS to prevent cross-ins. Furthermore, participants randomized to the PT only group will receive TENS unit after the primary outcome data is collected at day 60 which may also prevent cross-ins and provide equipoise. Primary analyses will be based on modified intention-to-treat which includes all participants with baseline (day 1) pre-TENS home activities.

Participants will be followed for 180 days with data collection from home at 1, 30, 60, 90, and 180 days following enrollment. Participants in the PT only group will also complete a brief assessment at day 65. The primary outcome variable will be assessed at day 60, which would be approximately at the completion of prescribed PT. Data collected at PT visits will include the types of treatments (CPT codes) and, at the clinic's usual intervals, visit type (in-person vs telemedicine), and the Patient-Specific Functional Scale (PSFS). All patient reported measures will be obtained at home via computer, tablet, or phone.

1.2 Questionnaires/Outcomes

The following questionnaires will be self-administered by participants:

- **Resting NRS for pain and fatigue:**11-point scale
- **Movement-evoked pain 5TSTS (Five times sit and stand) by NRS for pain and fatigue:** quantifies movement-evoked pain.
- **2016 FM Criteria**: diagnostic criteria for fibromyalgia.
- **PSFS (Patient Specific Functional Scale)** – quantifies activity limitation and measures functional outcomes
- **FIQR (Fibromyalgia Impact Questionnaire Revised)**: 21 items used to evaluate function, overall impact, and symptoms in patients with fibromyalgia.
- **MAF (Multidimensional Assessment of Fatigue)**:16 items measuring fatigue according to four dimensions: degree and severity, distress that it causes, timing of fatigue, and impact on various activities of daily living.
- **BPI (Brief Pain Inventory)**: 11 items measuring pain severity and interference with daily activities.
- **PROMIS-Physical function:** assesses information about physical activities.
- **PROMIS-Sleep & Sleep duration**: 8 items + a sleep duration question to assess sleep quality and quantity

• **PCS (Pain Catastrophizing Scale)**: 13 items measuring pain magnification, rumination, and helplessness.
• **PHQ-8 (Personal Health Questionnaire Depression Scale)**: 8 items measuring depressive disorders.
• **GAD-7 (Generalized Anxiety Disorder)**: 7 items measuring severity of various signs of generalized anxiety disorder.
• **RAPA (Rapid Assessment of Physical Activity)**: 9 items evaluating current level of physical activity.
• **TAPS (Tobacco, Alcohol, Prescription Medication, and other Substance use)**: 4 item screening tool for substance abuse.
• **PGIC (Patient Global Impression of Change)**: one item evaluating all aspects of patients' health and assesses if there has been an improvement or decline in clinical status.

1.3 Interventions and Duration

For the TENS+PT group, participants will be given TENS devices and electrodes following enrollment (signed consent) during their second PT visit. TENS will be applied to the upper and lower back with butterfly electrodes using the following parameters: mixed frequency (2-125Hz), strong but comfortable intensity, variable pulse duration from 100-250 microseconds. TENS will be applied for 2 hours per day at home when the patient is active and doing exercises and should be brought back to PT visits and used during PT treatment.

For the PT only group, participants will be given TENS units after the primary endpoint is collected at 60 days. The same parameters and instructions will be given to the participants as were given to the participants in the TENS+PT group. However, TENS instructions will be done virtually with a study team member rather than in person by their physical therapist.

1.4 Sample Size and Population

Initially, we proposed to enroll 600 participants from 24 physical therapy clinics: half of the clinics will be randomized to TENS+PT and the other half will be randomized to PT only. Based on a planned interim assessment of ICC for sample size recalculation, the target sample size was reduced to 450 participants (see section 9).

1.5 Inclusion Criteria

Age 18 or above

Clinician diagnosis of FM

Referred for land-based PT

Referred for treatment of FM or chronic pain (pain lasting more than 3 months)

Able to provide informed consent

Fluent in reading English

Willing to use TENS

1.6 Exclusion Criteria

Contraindications to TENS use (See Appendix 1 for Precautions and Contraindication references)

a. Pacemaker, defibrillator, implanted neurostimulator or implanted device
b. Epilepsy
c. Currently pregnant or plan to become pregnant in the next 6 months
d. Allergic reaction to patches with gel
e. Current treatment for cancer

Currently enrolled in another pain control study.

Use of TENS within the last 30 days.

Docusign Envelope ID: 15F39A01-5329-4125-A15A-B19595C72975

Clinically unstable medical or psychiatric issues

2. PRIMARY ENDPOINT

The primary objective of the study is to determine if addition of TENS to routine PT improves movement-evoked pain in patients with fibromyalgia (FM).

A primary effectiveness outcome will involve a comparison of the reduction of movement-evoked pain during a five times sit and stand test (5TSTS) measured by 11-point NRS from baseline to 60 days between the TENS+PT and PT only groups. The severity of pain will be measured on a 0 to 10 scale at each time where 0 is no-pain and 10 is worst pain imaginable. The baseline measurement will occur at home before the first instance of the patient applying the TENS device (or at parallel time for PT only patients). The 60-day measurement of movement-evoked pain will be obtained post-TENS after 30 minutes of TENS use for the TENS+PT group or at parallel time point for the PT only group. All outcomes will be collected by the participant at home. Movement-evoked pain during 5TSTS was measured in our previous randomized clinical trial, the FAST study, and the change in this measure was used to calculate the sample size for the current study.

3. SECONDARY ENDPOINTS

The secondary objectives of the study are to:

- *Determine if addition of TENS to routine physical therapy improves disease activity and symptoms, increases adherence to physical therapy, increases the likelihood of meeting patient specific functional goals, and reduces medication use.*

Change in the resting pain score from baseline (pre-TENS) to 60 days (post-TENS session) will be used as a secondary outcome variable. The severity of pain will be measured on a 0 to 10 scale at each time where 0 is no-pain and 10 is worst pain imaginable. Other secondary outcomes include change in the following domains:

o Pain interference (BPI)

o FM disease activity (FIQR)

o Resting fatigue (NRS)

o Movement-evoked fatigue (MEF)

o Multidimensional assessment of fatigue (MAF)

o Rapid assessment of physical activity (RAPA)

o Patient global impression of change (PGIC)

An exploratory outcome for patient-specific function using the patient-specific functional scale (PSFS) will be evaluated. Additional HEAL-specified measures and medications are located in the visit schedule. A brief description of the domains above can be found in section 1.2.

4. ENROLLMENT & RANDOMIZATION

Participants with fibromyalgia will be enrolled in outpatient physical therapy clinics: half of the clinics will be randomized to TENS+PT and the other half will be randomized to PT only. At each site, individualized PT treatment specified by the physical therapist alone or with TENS will be applied during each visit. Participants in the TENS+PT intervention clinics will receive TENS units and electrodes at baseline to be applied to the cervical and lumbar regions. They will take the units home and bring the units back to their PT clinic visits with instructions for use at home while active and during their exercises. The physical therapist at each clinic will complete routine documentation of treatment that will include the patient-specific functional scale (PSFS) at the intake visit and approximately monthly intervals.

This will be a cluster randomized pragmatic trial. A stratified randomization procedure by healthcare system and size of clinic will be used to randomize clinics to either TENS+PT or PT alone. Half of the clinics will be randomized to TENS+PT and the other half of the clinics will be randomized to PT only.

We hypothesize that using TENS in a PT setting is feasible and that FM patients using TENS in addition to their PT will have reduced movement-evoked pain and other symptoms and are more likely to reach their therapeutic goals and reduced medication use compared to those patients in the PT alone group.

5. TABULATIONS

All study participants who provide informed consent will be accounted for in this study. The number of randomized participants with baseline data and their study disposition will be reported overall, and by treatment group. A CONSORT diagram summarizing the final status of all study participants will be provided. The proportion of randomized study participants completing each visit will be summarized as in Table 5.1 below.

Table 5.1: Completeness of Study Data by Visit

Visit	TENS+PT (N = XX)	PT Only (N = XX)
BL (Day 1)	XX (XX%)	XX (XX%)
V01 (Day 30 ± 5)	XX (XX%)	XX (XX%)
V02 (Day 60 -5/+10) Primary Endpoint	XX (XX%)	XX (XX%)
V03 (Day 65 ± 5)	NA	XX (XX%)
V04 (Day 90 ± 5)	XX (XX%)	XX (XX%)
V05 (Day 180 ± 5)	XX (XX%)	XX (XX%)

Additional summary reports will describe:

- Number of study participants eligible, consented, and completed their day 1 home activities by site
- Reasons for exclusion
- Completeness of study visits, case report forms
- Protocol deviations
- Study Treatment (TENS) Suspensions
- Early Study Terminations
- Reportable Events
- Study Treatment Compliance
- Baseline participant data will also be summarized by treatment group (PT+TENS and PT only) with respect to important demographic characteristics. Distribution of numeric and categorical variables will be tabulated by treatment group and overall. Numeric variables will be summarized by the mean, standard deviation, minimum, and maximum. Categorical variables will be tabulated by frequency count and percentages. Variables that will be summarized include:
- Demographic Characteristics:
    - Age
    - Gender
    - Race
    - Ethnicity
    - FM Duration (Years)

Docusign Envelope ID: 15F39A01-5329-4125-A15A-B19595C72975

- FM Characteristics
  - o FM disease activity (FIQR)
  - o Opioid use at baseline
  - o Use of FM Medications

6. ANALYSIS POPULATIONS

6.1. Primary and Secondary Effectiveness Analyses (6 Month Treatment Period)

6.1.1. Intent-to-Treat (ITT) Population

For the primary outcome as well as all secondary outcomes during the 6 months follow-up period, all analyses will be performed consistent with the intention-to-treat (ITT) principle – per the recent JAMA Guide to Statistics and Medicine (Detry, 2014). With treatment randomization having occurred at the practice site level, the act of signing the consent automatically assigns a participant to the treatment arm the practice site was randomized to. However, it is possible that after signing consent that an enrolled participant will not show up at the PT clinic at day 1 or will not complete the home activities at day 1 where baseline measurements are obtained. Therefore, such a patient will be a "no-show" for the rest of the study duration. Thus, we will employ a modified ITT definition (mITT), such that the mITT population will include all participants assigned to a study arm that have completed baseline (day 1) home activities. All participants will be analyzed as-randomized, which means they will be analyzed based on the treatment to which they were randomized (whether the clinic is randomized to PT only or PT+ TENS), whether or not they remained compliant with respect to using their randomized TENS group assignment.

6.1.2. Per Protocol (PP) Population

To assess the sensitivity of key results and to obtain knowledge regarding the potential effects when the protocol was strictly adhered to, we will also replicate all key primary, secondary, and additional secondary effectiveness objectives using a per-protocol population. The per-protocol population includes the subset of all mITT participants who satisfy the following:

- Completed more than one PT appointment.
- And in addition, for those in the TENS group

  - Used TENS on at least 8 days within the first 30 days following day 1 homework assignment, and with an average of at least 30 minutes per day for the 30-day period (i.e. have accumulated a minimum of 15 hours of TENS use; 0.5 hr x 30=15 hours = 900 minutes),

    AND

  - Used TENS on at least 8 days during the subsequent 30 days (days 31–60), with an average of at least 30 minutes per day for the 30-day period (i.e. have accumulated a minimum of 15 hours of TENS use during days 31 to 60).

The above conditions for those in the TENS group to meet protocol assure continuous adherence of TENS use over the 60-day period.

Those in the TENS group who did not meet the definition of per protocol, 1) met criteria only for the first 30 days but not the second 30 days, or 2) provided TENS usage information but did not meet criteria for usage in both time periods, and 3) did not have any TENS usage information beyond the first day will be included in a dose response analysis of TENS.

7. PRIMARY ANALYSES

7.1 Primary Effectiveness Hypothesis: to determine if addition of TENS to routine PT improves movement-evoked pain (MEP) in patients with fibromyalgia (FM).

Primary objective: To conduct an effectiveness analysis comparing the TENS+PT and PT only groups.

The raw data for both treatment groups will be presented graphically, and summarized as in the following table:

Table 7.1: Descriptive Statistics of Movement-evoked Pain Over Time

Visit	TENS+PT (N = XX)	PT Only (N = XX)
Baseline Pre-TENS<br>Mean (SD)<br>(Min, Max)<br>Missing	XX.X (XX)<br>(XX.X, XX.X)<br>XX	XX.X (XX)<br>(XX.X, XX.X)<br>XX
Day 30 Post-TENS (Month = 1)<br>Mean (SD)<br>(Min, Max)<br>Missing	XX.X (XX)<br>(XX.X, XX.X)<br>XX	XX.X (XX)<br>(XX.X, XX.X)<br>XX
Day 60 Post-TENS (Month = 2)<br>Mean (SD)<br>(Min, Max)<br>Missing	XX.X (XX)<br>(XX.X, XX.X)<br>XX	XX.X (XX)<br>(XX.X, XX.X)<br>XX
Day 65 Post-TENS (Month 2)<br>Mean (SD)<br>(Min, Max)<br>Missing	NA	XX.X (XX)<br>(XX.X, XX.X)<br>XX
Day 90 Post-TENS (Month 3)<br>Mean (SD)<br>(Min, Max)<br>Missing	XX.X (XX)<br>(XX.X, XX.X)<br>XX	XX.X (XX)<br>(XX.X, XX.X)<br>XX
Day 180 Post-TENS (Month 6)<br>Mean (SD)<br>(Min, Max)<br>Missing	XX.X (XX)<br>(XX.X, XX.X)<br>XX	XX.X (XX)<br>(XX.X, XX.X)<br>XX

7.2 Analysis

To assess the effectiveness of TENS+PT compared to PT alone, we will compare the change in movement-evoked pain during 5TSTS from pre-TENS baseline to post-TENS at day 60. The null hypothesis can be evaluated based on an assessment of parameter estimates from a linear mixed effects model (LMM: Laird & Waire, 1982). This will be tested using LMM analysis with treatment group and movement-evoked pain level at baseline as fixed effects. Randomization will be stratified by the healthcare system and clinic size. The model will be adjusted for the fixed effect of clinic size. The proposed study involves multiple clinics under each healthcare system, which is a potential source of additional variation. Patients within a clinic tend to be correlated due to similarity of environment, e.g. because of getting PT treatment from the same set of physical therapists (Localio et al, 2001).Therefore, a random effect will be included for treatment nested within clinic, and within healthcare system.

For this analysis, the LMM can be written in the following manner:

$$Y_{ijk} = \beta_0 + \beta_1 X_{1i(jk)} + \beta_2 X_{2i(jk)} + \beta_3 T_{ijk} + a_{i(j)} + \epsilon_{\mathrm{ijk}}$$

where:

- Y_{ijk} is the change in movement evoked pain level from baseline to day 60 for the kth participant in i^th^ healthcare system and j^th^ clinic.
- β_0 is the common intercept.
- $X_{1i(jk)}$ is the movement evoked pain level at baseline for the kth participant .
- $X_{2i(jk)}$ if the participant was in the large clinical size strata at baseline, 0 otherwise.
- $T_{ijk} = 1$ if k^th^ participant in i^th^ healthcare system and j^th^ clinic is in the TENS+PT treatment group; = 0 otherwise.
- $a_{j(i)}$ is the random clinic effect within the ith healthcare system
- ϵ_{ijk} is a random error term for the kth participant in i^th^ healthcare system and j^th^ clinic.

Docusign Envelope ID: 15F39A01-5329-4125-A15A-B19595C72975

From the fitted model, the difference in mean change on pain between the two treatment groups will be assessed by the test for treatment effect. Accordingly, the main parameter of interest is β_3, which corresponds to the estimate of the difference in mean change in MEP between the TENS+PT vs PT only groups, controlling for baseline pain. *To assess the effectiveness of TENS+PT as measured by the change in MEP between baseline and Month 2 (Day 60), we employ the* LMM described above. Assuming the linear model provides the best fit, a test for the statistical significance of the mean difference between the two groups (TENS+PT vs. PT only) will be carried out as follows:

$$H_0: \beta_3 = 0 \quad vs. \quad H_A: \beta_3 \neq 0$$

Statistical significance for efficacy of TENS+PT vs. PT alone will be based on a two-tailed test at the 0.05 significance level with treatment effect summarized as mean difference with associated 95% confidence interval. If this hypothesis is not rejected, then we will conclude that there is not sufficient evidence to conclude that the *TENS+PT* treatment group is efficacious on MEP during the first two months of the treatment period. If the null hypothesis is rejected, figures will be utilized to assess which of the treatment groups shows significant differences over time with respect to the MEP scores. Each null hypothesis can be evaluated with the corresponding likelihood ratio test. Rejecting the null hypothesis implies that there is enough evidence to conclude that the use of TENS+PT is efficacious in reducing MEP compared to PT alone.

7.3. Missing Data for mITT Analyses

In the case of subject drop-out, reasons for subject drop-out will be recorded and compared between the two treatment groups. Subject characteristics and outcome measures collected prior to drop-out for those who drop-out will be compared to those who complete the study. The modified ITT analysis will include all consented participants in their assigned treatment arm that have completed baseline (day 1) home activities for the primary outcome. This will include those with missing follow-up data. Accordingly, the modified ITT analysis for the primary outcome measure of change in movement-evoked pain at day 60 includes participants with baseline data but with missing day 60 pain values, multiple imputation of the missing pain values will be conducted. Multiple imputation analyses assuming missing at random (MAR) will be performed using SAS PROC MI and SAS PROC MIANALYZE. To avoid imputation bias, all variables in the substantive analyses and, if feasible, other variables in the study database predictive of the missing values or influencing the cause of missing data will be included.

In addition to the MI analysis under MAR, since the data under analysis cannot distinguish if data is MAR or it is missing not at random (MNAR), sensitivity analysis will also be performed. Methods for sensitivity analysis such as marginal delta adjustment, conditional delta adjustment, reference-based controlled imputation, and other pattern mixture models will be considered. If needed, variables will be transformed to satisfy approximate normality before imputation and retransformed back to original scale.

7.4 Covariate Adjustments

It is expected that stratified randomization will lessen the need for covariate-adjusted analyses. However, in the event that adjusted analyses are necessary, a secondary comparison of the primary endpoint between groups will be made by expanding the linear mixed model to include covariates. Potential covariates include age, race, ethnicity, TENS dose/intensity, opioid use, and use of FM medications (for example, anti-depressants, α2-delta ligands). In addition, opioid use at baseline as a possible effect moderator of TENS will be examined by including an opioid*treatment interaction in the model. If found to be significant, then secondary analyses to test for TENS effectiveness by opioid status will be performed with p-values adjusted using Bonferroni's method for multiple comparisons. Statistical significance for effectiveness of TENS+PT vs. PT alone will be based on a two-tailed test at the 0.05 significance level with treatment effect summarized as mean difference with 95% confidence interval.

8. SECONDARY ANALYSES

Analyses for secondary outcome measures will be performed using the analyses methods described in section 7 above. The list of the secondary outcomes is in section 3 above.

9. SAMPLE SIZE JUSTIFICATION

The required sample size was calculated to assess the effect of TENS+PT versus PT alone on the primary outcome of change from baseline to 2-month visit in pain after PT using two-independent samples t-test with inflation factor of [1+(m-1)*ICC] applied to account for average cluster size (m) and intra-cluster correlation (ICC). From the FAST study, there was a standard deviation of 2 for change in movement-evoked pain for the 5TSTS test. There was a greater decrease in pain with active- TENS compared to placebo-TENS of 1.3 (95% CI: 0.34, 2.2) for movement-evoked pain during 5TSTS. Compared to no-TENS, there was a greater decrease in pain with active-TENS of 1.8 (95% CI: 1.0, 2.8) for movement-evoked pain during 5TSTS. For this cluster randomized pragmatic trial comparing TENS+PT vs. PT alone, sample size was determined such that the statistical test at the 0.05 significance level will be able to detect a difference of at least 1.0 in mean change in pain with 80% power. Participants randomized to a treatment arm will be clustered by clinic, since PT clinics will be randomized for ease of implementation. Thus, an estimate of the intra-cluster correlation (ICC) was needed for the sample size calculation. With no prior estimate of ICC, the required sample size per treatment arm (or participants per clinic) for the desired detectable mean difference of 1.0, assuming SD=2.0, was calculated for combinations of ICC values and number of clinics per treatment arm; see Table 1 for ICC values for a sample size <300 participants per treatment arm. A pilot study examining a non-pharmacological intervention for pain by our group showed an ICC of 0.01, DeBar and colleagues used an ICC of 0.002 for sample size calculation for a chronic pain population, and Adams et al. showed widely varying ICCs between data sets, but the majority of patient-reported outcomes were below 0.095 ICC. Therefore, the study sample size was originally calculated for ICC value between 0.12 and 0.14 which resulted in a conservative sample size estimate of 456 total participants (228 per treatment arm) with complete data, for 9 to 12 clinics per arm. Table 9.1 shows the number of participants per clinic for each treatment arm, for specified ICCs and range of 9 to 12 clinics per treatment arm (C), to detect a mean difference of at least 1 (in NRS scale of 0 to 10) in pain at the 0.05 significance level with 80% power. Numbers in black are the sample size needed for combinations of ICCs and number of clinics for n≤228/arm while the tan numbers are for n>228 per treatment arm.

For example, if the ICC is 0.14, to reach 80% power with 12 clinics in each arm, complete data will be needed from 19 patients per clinic, 228 patients in each arm: 456 patients total. The dropout rate in the previous randomized clinical trial from our group, the FAST study, was 12% at 30-day follow-up. Since follow-up is 60 days in the proposed study, a higher drop-out rate is expected. The drop-out rate includes those patients who complete the informed consent form but do not complete the baseline assessment as a part of day 1 home activities, as well as those patients who start the study and dropout prior to day 60. To account for up to 24% drop-out rate, a total of 600 participants (300 per arm) will be enrolled. It should be noted that expected enrollment will average 25 patients per clinic for the scenario with 12 clinics per treatment arm, with some variability expected in the number of patients enrolled from each clinic. The number of patients enrolled from each clinic will be capped at 30 patients to allow for enrolling up to 20% more than originally planned.

An interim ICC recalculation will be conducted after the first 200 participants have been enrolled (approximately 1/3 of the sample) for the purpose of determining ICC for the primary outcome measure. This analysis to assess the primary outcome variable will be done for the purpose to only calculate the ICC value using the change in pain scores without regard to group assignment and will not require unblinding of randomization. Therefore, it does not have an impact on the type I error rate and there is no change in our planned analysis on our statistical analysis plan. This analysis will only be used to determine if the final number of subjects per site should be changed based on the ICC value. There are no stopping rules.

Table 9.1: Total study sample size with varying ICC, number of clinics per arm and subjects per clinic.

Docusign Envelope ID: 15F39A01-5329-4125-A15A-B19595C72975

ICC	ICC values by clinics (C), per treatment arm; subjects per clinic (total subjects)			
	C=9	C=10	C=11	C=12
0.050	11 (99)	9 (90)	8 (88)	7 (84)
0.100	23 (207)	16 (160)	13 (143)	11 (132)
0.110	30 (270)	20 (200)	15 (165)	12 (144)
0.120	--	25 (250)	17 (187)	14 (168)
0.125	--	28 (280)	19 (209)	14 (168)
0.130	--	--	21 (231)	16 (192)
0.135	--	--	24 (264)	17 (204)
0.140	--	--	27 (297)	19 (228)
0.145	--	--	--	21 (252)
0.150	--	--	--	23 (276)
0.155	--	--	--	--

The original sample size calculation conservatively assumed the ICC values to change between 0.12 and 0.14, subjects to be enrolled from 12 clinics in each group, and approximately equal number of subjects to be enrolled from each clinic. During the conduct of the study, as clinics were recruited, some other clinics were deactivated. It was also observed that there was variability in the number of patients enrolled per clinic. This observed variability required the need to account for the variation in the number of enrolled participants per clinic in the sample size calculation. For this purpose, the coefficient of variation (CV), which is the ratio of standard deviation (sd) to the mean (CV = sd / mean), was used in the sample size calculation to represent variation in enrollment per clinic

The number of active clinics at the time of the ICC recalculation was 28, where 26 clinics (13 in each treatment) had at least one participant with a day 1 assessment. The observed CV was 0.6.

Plans for sample size recalculation were discussed with Drs. Liz Turner and Patrick Heagerty from the NIH Collaboratory Biostatistics Core in November 2022. This meeting resulted in two recommendations: 1) recalculating the ICC value based on the adjusted model, consistent with the final analyses. 2) Calculating the ICC and the Jackknife estimate of standard error (SE) of ICC, and calculating the sample size using both the current estimate of ICC and ICC + 1SE as a conservative estimate.

These recommendations were included in the implementation of the interim ICC estimation and sample size recalculation. With 220 enrolled subjects, the estimate of the ICC based on the adjusted model, consistent with the SAP, was 0.05 with the Jackknife estimate of the SE of ICC of 0.07. Taking into account the variability in enrollment numbers per clinic (CV = 0.6) and using 13 clinics/group, the sample size was estimated at 450 enrolled subjects to obtain a final number of 342 participants completing day 60 based on a 24% dropout rate. This sample size provides >90% power for ICC = 0.05, 81% power for ICC = 0.10, and 78% power for ICC of 0.12 (ICC + 1SE = 0.05 + 0.07). The study sample was updated to 450, assuming an ICC value of 0.10. The sample size recalculation was presented to NIAMS and the DSMB. Both NIAMS and the DSMB recommended approval of the change in sample size.

10. SAFETY MONITORING

10.1 Adverse Events

All aspects of the study will be monitored by authorized individuals in compliance with Good Clinical Practice (GCP) and applicable regulations. Refer to the Safety Management Plan for additional details.

An adverse event in this study will be defined as any unfavorable and unintended sign or symptom temporally associated with the use of the TENS units, falls while performing the Sit and Stand Test during Research Homework or physical therapy exercise, changes in health status that would impact ability to use the

intervention TENS, or hospitalizations/Emergency Department visits. Pain associated with physical therapy treatment may worsen during the study but will not be classified as an AE.

Anticipated adverse events with TENS use include:

- Skin rash or irritation from electrodes
- Anxiety, itchiness, nausea or pain with TENS

10.2 Assessment and Recording of Adverse Events

The study team may contact the participant when an adverse event has been reported to collect further information on the AE, if needed. The team will determine if the adverse event is serious, related to TENS treatment, and if any action needs to be taken, for example, discontinue TENS use, or if further reporting to regulatory authorities is necessary.

Pregnancy: Pregnancy itself is not regarded as an AE. However, TENS is not recommended for use during pregnancy and the participant will be withdrawn from treatment and the outcome of all pregnancies that occur during paternal or maternal exposure to study device (spontaneous miscarriage, elective termination, normal birth or congenital abnormality) must be followed up and documented even after the participant has been withdrawn from treatment.

10.3 Management of Unanticipated Problems

Unanticipated problems will be defined as unexpected adverse clinical symptoms related to TENS or electrode use. These will be reported as “other” TENS-related AE on the CRF and will be reviewed at each DSMB meeting. If unanticipated TENS-related AE are uncovered that occur in >5% of enrolled participants, the consent form will be modified, and the AE will be specifically queried on the AE CRF going forward. Participants who have completed the study will be notified of any newly recognized TENS-related AE.

Docusign Envelope ID: 15F39A01-5329-4125-A15A-B19595C72975

REFERENCES

Detry MA and Lewis RJ (2014). "The Intention-to-Treat Principle: How to Assess the True Effect of Choosing a Medical Treatment," *Journal of the American Medical Association*, 312(1): 85-86.

Laird NM and Ware JH (1982). "Random-Effects Models for Longitudinal Data," *Biometrics*, 38(4): 963-974.

Little RJA and Rubin DB (2002). *Statistical Analysis with Missing Data, Second Edition*. New York: Wiley.

Localio AR, Berlin JA, Ten Have TR, and Kimmel SE (2001). "Adjustments for Center in Multicenter Studies: An Overview," *Archives of Internal Medicine*, 135(2): 112-123.

Senn S (2013). "Seven Myths of Randomization in Clinical Trials," *Statistics in Medicine*, 32: 1439-1450.

Verbeke G and Molenbergs G (2000). *Linear Mixed Models for Longitudinal Data*. New York: Springer.

Wenqiang C, Lonskaya I, Hebron ML, Ibrahim Z, Olszewski RT, Neale JH, and Moussa CE (2014). "Parkin-Mediated Reduction of Nuclear and Soluble TDP-43 Reverses Behavioral Decline in Symptomatic Mice," *Human Molecular Genetics*, 23(18): 4960-4969.

Wyse RK, Brundin P, and Sherer TB (2016). "Nilotinib – Differentiating the Hope from the Hype," *Journal of Parkinson's Disease,* 6(3): 519-522.

Heidari F, Afshari M, Moosazadeh M. Prevalence of fibromyalgia in general population and patients, a systematic review and meta-analysis. Rheumatol Int 2017; 37:1527-39.

Okifuji A, Hare BD. Management of fibromyalgia syndrome: review of evidence. Pain Ther 2013; 2:87-104.

Vincent A, Whipple MO, McAllister SJ, Aleman KM, St Sauver JL. A cross-sectional assessment of the prevalence of multiple chronic conditions and medication use in a sample of community-dwelling adults with fibromyalgia in Olmsted County, Minnesota. BMJ Open 015;5: e006681.

Qaseem A, Wilt TJ, McLean RM, Forciea MA, Clinical Guidelines Committee of the American College of P. Noninvasive Treatments for Acute, Subacute, and Chronic Low Back Pain: A Clinical Practice Guideline from the American College of Physicians. Ann Intern Med 2017; 166:514-30.

Macfarlane GJ, Kronisch C, Atzeni F, et al. EULAR recommendations for management of fibromyalgia. Ann Rheum Dis 2017;76: e54.

Dowell D, Haegerich TM, Chou R. CDC guideline for prescribing opioids for chronic pain-United States. MMWR Recomm Rep2016:1-49.

Vance CG, Dailey DL, Rakel BA, Sluka KA. Using TENS for pain control: the state of the evidence. Pain Manag 2014; 4:197-209.

Sluka KA, Walsh DM. Transcutaneous Electrical Nerve Stimulation and Interferential Therapy. In: Sluka KA, ed. Pain Mechanisms and Management for the Physical Therapist. 2nd Edition ed. Philadelphia: Wolters Kluwer; 2016:203-23.

Bjordal JM, Johnson MI, Ljunggreen AE. Transcutaneous electrical nerve stimulation (TENS) can reduce postoperative analgesic consumption. A meta-analysis with assessment of optimal treatment parameters for postoperative pain. Eur J Pain 2003; 7:181-8.

Dailey DL, Vance CG, Rakel BA, Zimmerman MB, Embree J, Merriwether EN, Geasland KM, Chimenti R, Williams J, Golchha M, Crofford LJ, Sluka KA. A Randomized Controlled Trial of TENS for Movement-Evoked Pain in Women with Fibromyalgia. Arthritis & rheumatology (Hoboken, NJ). 2019 Nov 18 doi: 10.1002/art.41170. [Epub ahead of print]

Docusign Envelope ID: 15F39A01-5329-4125-A15A-B19595C72975

Wolfinger R , O'Connell M (1993) Generalized linear mixed models: a pseudo-likelihood approach . Journal of Statistical Computation and Simulations, 48, 233-243 .

Google Scholar | Crossref Open URL

Zeger SL , Karim MR (1991) Generalized linear models with random effects; a Gibbs sampling approach . Journal of the American Statistical Association, 86, 79-86 .

Google Scholar | Crossref | ISI Open URL

Zeger SL , Liang K , Albert PS (1988) Models for longitudinal data: a generalized estimating equation approach . Biometrics, 44, 1049-1060 .

Google Scholar | Crossref | Medline | ISI Open URL
