# Supplementary material for: Transcutaneous Electrical Nerve Stimulation and Pain With Movement in People With Fibromyalgia: A Cluster Randomized Clinical Trial
Source: JAMA Netw Open. 2026 Mar 27;9(3):e262450. doi: 10.1001/jamanetworkopen.2026.2450 (PMC13032160; doi:10.1001/jamanetworkopen.2026.2450)
Supplement: Supplement 2. — eMethods. Additional methods eTable 1. Demographic and outcome data for those meeting and not meeting the ACR Diagnostic Criteria eTable 2. Cluster-level consort information eTable 3. Results of different imputation methods on movement-evoked pain eTable 4. Sensitivity analysis for those who met the fibromyalgia diagnostic criteria (fibromyalgia-positive) and those who did not meet criteria at baseline (fibromyalgia-negative). eTable 5: Sustained effect of TENS use in fibromyalgia eTable 6. Per protocol analysis eTable 7. Participant experience with TENS eTable 8. Adverse events (AE) related to TENS eTable 9. Adverse events unrelated to TENS eFigure. Per protocol analysis eReferences [file jamanetwopen-e262450-s002.pdf]

Supplemental Online Content

Dailey DL, Vance CGT, Van Gorp BJ, et al. Transcutaneous electrical nerve stimulation and pain with movement in people with fibromyalgia: a randomized clinical trial. *JAMA Netw Open*. 2026;9(3):e262450. doi:10.1001/jamanetworkopen.2026.2450

eMethods. Additional methods

eTable 1. Demographic and outcome data for those meeting and not meeting the ACR Diagnostic Criteria

eTable 2. Cluster-level consort information

eTable 3. Results of different imputation methods on movement-evoked pain

eTable 4. Sensitivity analysis for those who met the fibromyalgia diagnostic criteria (fibromyalgia-positive) and those who did not meet criteria at baseline (fibromyalgia-negative).

eTable 5: Sustained effect of TENS use in fibromyalgia

eTable 6. Per protocol analysis

eTable 7. Participant experience with TENS

eTable 8. Adverse events (AE) related to TENS

eTable 9. Adverse events unrelated to TENS

eFigure. Per protocol analysis

eReferences

This supplemental material has been provided by the authors to give readers additional information about their work.

© 2026 Dailey DL et al. *JAMA Network Open*.

eMethods. Additional methods

Clinics

In 2019, outpatient physical therapy clinics in the Midwest were contacted by the Principal Investigators from both the University of Iowa and Vanderbilt University. Outpatient physical therapy (PT) clinics were eligible for participation if they treated 60 or more patients with fibromyalgia and/or chronic pain in the last year and willing to participate. We selected 36 clinics from 6 healthcare systems in the Midwest. Over the course of the study, 36 clinics from 6 healthcare systems across 7 states were activated, 11 clinics were deactivated, and 28 clinics contributed to the final dataset. Clinics were deactivated by clinic request or limited performance in enrollment. Clinics participated in the study for an average of 29 months (range from 2 to 43 months). Physical therapists were licensed to practice in their state, employed by the enrolling clinic and willing to participate in the study.

Clinic recruitment and onboarding occurred before, during and after the COVID pandemic, involving in-person and virtual meetings with clinic leaders and managers. These meetings provided an overview of the study, outlined the clinicians' roles, and introduced the study intervention, Transcutaneous Electrical Nerve Stimulation (TENS). Following identification of clinics and final approval by clinic leaders, the study team met virtually or in-person with the clinicians at each individual's clinic. This meeting with the physical therapy clinicians focused on providing an overview of the study and questions about clinic workflow, knowledge and use of TENS, and treatment approaches for individuals with chronic pain or

© 2026 Dailey DL et al. *JAMA Network Open*.

fibromyalgia (FM). Clinic managers or supervisors completed surveys regarding the number of patients with chronic pain over the last year, number of physical therapists and physical therapy assistants. Final approval by individual clinic leaders was based on their willingness to participate in the study.

All enrolling physical therapists received training in Good Clinical Practice and Protection of Human Subjects. Clinic training was completed both in person or virtually and included study training on communication, intervention training, TENS and iPad training, and study flow. The total clinic training time was 2-2.5 hours. Written materials included a clinician manual, a participant manual, TENS use practice, TENS application manual, recruitment materials. Clinicians were provided study contact information including phone and email contacts, as well as website information. Booster training sessions and regular clinic visits both virtually and in-person ensured continued engagement and adherence to the study protocol.

Study Team

The Clinical Trials and Data Management Center at UI served as the Data Coordinating Center. Our team employed individuals with lived experience of fibromyalgia and these individuals were involved in all aspects of the study design. We specifically consulted individuals with fibromyalgia in testing the design and development of instructions of the primary outcome measure, movement-evoked pain, to be performed at home. All marketing, patient-facing materials, and community engagement materials were

© 2026 Dailey DL et al. *JAMA Network Open*.

developed and reviewed by study team members who have lived experience with fibromyalgia. In addition, we consulted practicing physical therapists from our enrolling clinics in the design of the study, screening and enrollment procedures, and methods to modify workflow. We also worked with local communities where the physical therapy clinics were located to enhance awareness and enrollment and consulted practicing physical therapists in the design of flyers, postcards and social media advertisements.

Intervention Design

We designed the study as a cluster-randomized trial with clinics randomized to PT+TENS or PT-Only. The TENS intervention used the same parameters as our prior randomized controlled clinical trial which showed a significant effect of TENS compared to both a placebo group and to a no-TENS group[1]. As the prior randomized controlled trial showed efficacy against placebo for reductions in movement-evoked pain, we chose to compare the effectiveness of TENS in a real world setting against those who did not receive TENS. We chose to implement the intervention into outpatient physical therapy clinics whose primary intervention is exercise for fibromyalgia, and ask subjects to use while performing physical therapy exercises in clinic and at home and when active. Our primary goal was to determine if TENS would work individuals with a clinical diagnosis of fibromyalgia during activity. We did not use a placebo comparison given results of our prior randomized controlled trial and to more fully mimic how the intervention would be used in a clinical setting. We do expect the effectiveness of TENS will reflect both its non-specific and specific effects, which are both clinically relevant to the intervention.

© 2026 Dailey DL et al. *JAMA Network Open*.

After Day 60, the randomized phase, the PT-Only group was provided TENS and used for the duration of the study through Day 180. This was used to enhance retention of the PT-Only group in the study. We used a similar approach in our prior randomized controlled trial, FAST to enhance retention. Study manuals were developed for the PT-Only Group and the PT+TENS group to orient the to the study. These manuals included information on funding, goals, design, requirements, physical therapy visits, RedCap, and contact information. THE PT+TENS manual also included information on TENS. Both groups were told there were 2 study groups, that both groups were important to the study and that the goal of the study to test if TENS worked in a real-world clinical setting to reduce movement pain and improve function. The No TENS group was also told that their data during the first 60 days was critical to compare differences between groups if adding TENS to physical therapy provides better outcomes than those who just receive physical therapy, and that they would receive TENS after completing Day 60.

The intervention was provided by a licensed physical therapist. We trained 79 physical therapists in the PT+TENS group and 82 in the PT-Only group. We also trained 15 PT assistants (5 in PT+TENS and 7 in PT-Only group), 12 office staff (5 in the PT+TENS group, 7 in the PT-Only group), 1 student physical therapy assistant in the PT-Only group, and 2 Occupational therapists in the PT-Only who assisted with other study procedures. The study did not collect information on the treating physical therapist for each participant.

© 2026 Dailey DL et al. *JAMA Network Open*.

TENS Instruction

TENS instruction for physical therapists covered indications for use, contraindications for use, and care of the TENS units, skin, and electrodes. For participants, the manual provided contact information for the study team to troubleshoot problems with the TENS units and electrodes as needed. Participants were provided TENS units which they kept at the end of the study and 6 months of electrodes.

Participants

Initial inclusion and exclusion criteria are as follows. Inclusion Criteria included adults 18 or above with a clinician diagnosis of fibromyalgia, referred for land based physical therapy and for treatment of FM, chronic neck pain, or chronic back pain, able to provide consent, fluent in reading English and willing to use TENS. Exclusion criteria were contraindications to TENS: Pacemaker, defibrillator, implanted neurostimulator or implanted device, epilepsy, currently pregnant or plan to become pregnant in the next 6 months, allergic reaction to patches with gel, current treatment for cancer, currently enrolled in another pain control study, use of TENS within the last 6 months, or clinically unstable medical or psychiatric issues. The protocol was changed to enhance enrollment by expanding the inclusion criteria to those referred for fibromyalgia or any chronic pain condition on 4/29/2023 and reducing the exclusion criteria to use of TENS within the last 30 days 9/9/2021.

© 2026 Dailey DL et al. *JAMA Network Open*.

Since the clinics were cluster randomized to the intervention, participants were randomized to be in the TENS+PT or PT-Only group based on clinic allocation. The study PIs, which included the safety officer, were blinded to participant intervention group.

Recruitment and Retention

Recruitment and retention methods were directed to both potential and enrolled participants, and enrolling physical therapists. The clinical study team was comprised of three groups: a clinic direct team, a patient direct team, and a community engagement team. The clinic direct team was responsible for communication with clinics, clinicians, clinic liaisons, supervisors, managers, and chief executive officers (CEOs). The clinic direct team also conducted study training, facilitated Institutional Review Board (IRB) human subjects training and equipment needs, scheduled virtual or onsite visits with clinicians, communicated study updates and changes, and created weekly clinic specific reports about screening and enrollment. The patient direct team was responsible for assisting with participant communication. This team monitored patient screening, eConsent completion, and research homework completion. This team communicated with participants via telephone, email, and/or text. They communicated reminders for research homework, provided explanations of the study, troubleshot equipment issues, and answered participant questions through the study website, telephone, and/or email. The patient direct team logged all communication with participants or therapists. The community engagement team directly targeted outreach to the community using a variety of techniques which have been outlined in a previously published manuscript[2].

© 2026 Dailey DL et al. *JAMA Network Open*.

There was no on-site coordinator at any of our PT sites; however, each site had a study team member that directly communicated with the clinic. The study team member visited each clinic every 1-3 months in-person and held monthly meetings virtually. Quarterly meetings with the healthcare system liaison were held to troubleshoot recruitment. Discussions with CEOs and owners were held to discuss enrollment incentives and distribution or credit provided to enrolling PTs and clinics.

We implemented numerous methods to enhance recruitment and retention. Using input from clinics, we made a “tips” sheet outlining key activities for each enrolling physical therapist. Clinics received monetary incentives for each enrollment and there were non-monetary incentives for participating PTs when they reached designated enrollment targets. All non-monetary incentives were branded with the FM-TIPS logo and contact information. Letter templates to send to their referring providers and other providers in their area were provided to clinics and the study team made visits to local referring provider offices with packets of study information. Marketing materials were developed and placed on the clinic website informing potential subjects about the study. Brochures and posters were developed and placed in clinics, community settings and referring provider offices. Contests for enrollment and recruitment were held for each healthcare system with food incentives as rewards. The study PIs sent a study update e-mail to each clinic with their current recruitment status and targeted goals every year. The study team provided continuing education seminars regularly throughout the enrollment period.

© 2026 Dailey DL et al. *JAMA Network Open*.

Community talks on pain management, physical therapy, and fibromyalgia were developed in partnership with the local clinics. The community engagement team attended fairs and farmers markets to enhance study awareness. Postcards were mailed to individuals in rural communities promoting the study. The study was also advertised with targeted Facebook and Google ads primarily in rural communities.

For participants, we implemented automatic reminders (email and text), timely e-mails, and phone contact with participants to ensure data completion with a focus on passing screening, enrollment and Day 1 homework, Day 30, Day 60, Day 90 and Day 180 homework. Participants received free TENS units, 6 months of electrodes and financial reimbursement for their time.

Collection of Adverse Events

Adverse events were self-reported by the participant at the time of data collection on day 30, 60, 90, and 180 on a case report form developed by the study team. We collected adverse events related to the intervention (TENS) as well as information on emergency room visits and hospitalizations, falls during the sit and stand test, and falls during physical therapy home exercise. The safety officer reviewed adverse events monthly and more frequently on an as needed basis. Participants also reported on new health conditions that could be contraindications to TENS. If they reported “yes” to any potential contraindication the study officer followed up with the participant to determine the risk of continuing the

© 2026 Dailey DL et al. *JAMA Network Open*.

intervention. No participants had to stop the intervention because of a new contraindication.

Participant Equipment, Supplies, and Instruction

Each clinic received two TENS units, chargers, and electrodes for dedicated use in the clinic and retained them at the end of the study. Participant manuals specific to the arm of the study were prepackaged and shipped in participant bags to use for participant education. For the TENS+PT group, this bag contained a study manual, TENS units, chargers, electrodes, TENS manual, TENS application manual, and study contact information. For the PT-Only group, the participants received a bag with their study manual and contact information. The PT-Only group were mailed TENS units, electrodes, TENS manual, and TENS application manual after completion of Day 60 research homework and received virtual training by a study team member.

FM-TIPS created a study website with information for participants and clinicians. Information on the site included information about the aim of the study, education about TENS and FM, a supply ordering form for participants and a separate one for enrolling clinics, and inquiry form for potential participants, and contact information for email, text or telephone.

Statistical Analysis

© 2026 Dailey DL et al. *JAMA Network Open*.

Sensitivity analyses were conducted to evaluate the robustness of the results under different missing data mechanisms. For outcomes with missing values at Day 60 (including movement-evoked pain, resting pain, resting fatigue, and movement fatigue) the missing data were imputed simultaneously using multiple imputation via Monte Carlo Markov Chain (MCMC) methods. Several additional imputation methods were considered for the aforementioned outcomes, including analysis using only observed data, imputation of Day 60 using baseline data only, last observation carried forward (LOCF), incorporating Day 30 post-PT, Day 1 post-PT, and Day 1 pre-PT data when available, and worst-case scenario imputation, where missing Day 60 values were replaced by the patient's worst observed pain score (highest value) up to Day 60 in the TENS+PT group, and the best observed pain score (lowest value) in the PT-only group. Finally, we also considered a longitudinal approach with similar random effects, accounting for temporal correlation using maximum likelihood estimation. Specifically, we modeled change from baseline, including treatment status, time (Day 30 and Day 60), and their interaction as fixed effects in the models. Treatment effect p-values at Day 60 obtained from Wald tests were reported. A similar model and an LMM based only on observed data were used for other secondary outcomes. Additionally, a one-dimensional tipping point analysis was conducted to assess the robustness of the treatment effect for the four outcomes (movement-evoked pain, resting pain, resting fatigue, and movement fatigue) by systematically shifting the mean of imputed Day 60 values in the opposite direction of the observed effect, identifying the threshold at which the interaction term became nonsignificant.

© 2026 Dailey DL et al. *JAMA Network Open*.

All analyses were performed in accordance with the intention-to-treat principle, which means that participants were analyzed based on the treatment to which they were randomized, regardless of their compliance with the use of their assigned TENS treatment. To assess the sensitivity of primary results and to obtain knowledge regarding the potential effects when the protocol was strictly adhered to, we replicated the primary analyses on the per-protocol population. The per-protocol population includes the subset of all mITT (defined as those who completed baseline assessments) participants who used TENS at an adequate dose and completed more than one PT visit. An adequate dose was defined as using 8/30 days and an average of 30 min/day (total of 900min/month). Groups were defined as follows: Group 1 used TENS at the adequate dose for both the first 30 and the second 30 days; Group 2 used TENS at an adequate dose for the first 30 days, Group 3 used TENS less than the adequate dose. These groups were compared to PT-Only group which did not use TENS for the first 60 days. All statistical analyses were performed using SAS version 9.4 (SAS Institute Inc., Cary, NC).

Model Fit and Residual Diagnostics

Although the numerical rating scale is ordinal by design, change scores derived from Likert-type scales with multiple response categories are commonly analyzed as continuous outcomes. Linear models have been shown to be robust to moderate departures from normality and ordinality, especially when the scale has ≥7 response categories (as in our case), the distribution of observed values spans much of the scale, and the analysis focuses on mean differences. Besides, for our dataset, residual diagnostics for the mixed-

© 2026 Dailey DL et al. *JAMA Network Open*.

effects models (including residual–fitted and Q–Q plots) did not indicate meaningful violations of model assumptions (except for RAPA), supporting the adequacy of the chosen approach. This analytic strategy is well supported in the methodological literature and widely used in clinical research, which shows that parametric tests are sufficiently robust to yield largely unbiased estimates that are close to "the truth" for Likert-scale responses[3,4]. Since the RAPA scores are on a ≤5 scale, based on the reviewer comments, we decided to perform additional sensitivity analyses treating RAPA as a binary outcome. We added supplemental analyses modeling the RAPA scores using generalized linear mixed modeling with logit link [RAPA1: 0-2 (no weekly physical activity) vs 3-5 (weekly physical activity), and RAPA2: 0 (no activities to increase flexibility of strength once a week) VS 1-3 (activities to increase flexibility of strength once a week)] at day 60. These additional results were consistent with the original RAPA results, showing no differences between treatment groups.

Data Sharing

We have registered our study on ClinicalTrials.gov and the NIH HEAL Initiative, HEAL Data Platform. We indicated our intent to publicly share the final participant-level clinical study data with the Inter-University Consortium for Political and Social Research (ICPSR) repository for data archive. We have entered study-level information on the CEDAR MetaData Center. Prior to sharing, all data will be de-identified to ensure HIPAA compliance. Operational data and data with comment fields will not be shared as they may reveal the identity of the participants. We will provide all necessary documentation (e.g.,

© 2026 Dailey DL et al. *JAMA Network Open*.

Protocol, Manual of Procedures/Operations, Statistical Analysis Plan, Data Dictionary; Informed Consent Form Template, Annotated Case Report Forms (CRF), CRF Mapping Spreadsheet; Scoring Document for the Secondary Endpoints; Read Me file) to the ICPSR Repository along with the final datasets. The data dictionary will be provided, including variable names, descriptions, formats, and codes to ensure that other users can efficiently and accurately use the data without misinterpretation or misuse. Study metadata will be available in the HEAL central catalog and CEDAR entry.

© 2026 Dailey DL et al. *JAMA Network Open*.

eTable 1. Demographic and outcome data for those meeting and not meeting the ACR Diagnostic Criteria

	ACR Criteria Negative (n=91)	ACR Criteria Positive (n=286)	P-value
**Demographics**			
Age (yrs, mean ± SD)	54.6 ±14.7	52.7 ± 15.2	0.284
Sex (%, Female)	86 (93%)	261 (92%)	1.0
Ethnicity			0.276
Hispanic/Latino (number, %)	6 (7%)	20 (7%)	
Not Hispanic or Latino (number, %)	83 (90%)	240 (84%)	
Unknown/Not Reported (number, %)	5 (5%)	26 (9%)	
Race			.906
American Indian/Alaska Native (number, %)	0 (0%)	3 (1%)	
Asian (number, %)	0 (0%)	3 (1%)	
Black/African American (number, %)	6 (7%)	26 (9%)	
Multiracial (number, %)	1 (1%)	6 (2%)	
Native Hawaiian/Pacific Islander	0 (0%)	2 (<1%)	
White (number, %)	81 (88%)	230 (80%)	
Unknown/Not Reported (number, %)	6 (7%)	16 (6%)	
Education (%, High School or less)	28 (30%)	120 (42%)	0.071
Income (%, <$50,000 per year)	39 (42%)	132 (46%)	0.291
**Fibromyalgia (FM) Measures (Mean ± SD)**			
Fibromyalgia impact (FIQR, 0-100)	44.2 ±16.5	61.5 ± 15.5	0.001
Widespread pain (FM Diagnostic Criteria 2016, WPI, 0-19)	4.8 ± 2.3	10.8 ± 3.3	0.001
Somatic symptoms (FM Diagnostic Criteria 2016, SSS, 0-12)	5.4 ± 1.9	8.2 ± 1.9	0.001
Polysymptomatic Distress Scale (FM Diagnostic Criteria 2016, PSD, 0-31)	10.4 ± 2.3	19.0 ± 4.1	0.001
**Baseline Measures of Outcome Variables (Mean ± SD)**			
Movement Evoked Pain (MEP, NRS, 0-10)*	4.5 ± 2.4	5.9 ± 2.0	0.001
Pain at rest (NRS, 0-10)*	4.5 ± 2.3	5.8 ± 1.8	0.001
Pain interference (BPI, 0-10)*	4.8 ± 2.2	6.5 ± 1.9	0.001
Pain severity (BPI, 0-10)*	5.0 ± 1.9	6.2 ± 1.6	0.001
Fatigue at rest (NRS, 0-10)*	4.4 ± 2.4	6.3 ± 2.0	0.001
Fatigue with movement (NRS, 0-10)*	4.6 ± 2.4	6.5 ± 2.0	0.001
Activity average (PSFS, 0 to 10)†	5.1 ± 2.3	4.2 ± 2.5	0.009

© 2026 Dailey DL et al. *JAMA Network Open*.

eTable 2. Cluster-level consort information. *some of the clinics deactivated at the request healthcare system request enrolled subjects and were included in the analysis

	**PT-Only**	**PT+TENS**
Number Activated	18	18
Number Enrolling at least one participant	15	14
Number with one participant that reached modified intention-to-treat (mITT)	15	13
Deactivated-low-enrollment*	2	2
Deactivated-HCS request	4	3
Number months individual clinics active - enrolling clinics (mean, range)	36.7 (18-46)	32.5 (11-46)
Number clinics activated per healthcare system	3.6 (1-5)	3.6 (1-5)
Large clinics	8 (44.4%)	6 (33.3%)
Rural clinics – activated group	9 (50%)	7 (39%)
Rural clinics – enrolling group	7 (47%)	5 (35%)
Number PTs trained per clinic (mean, range) (mean, range)	4.4 (1-12)	4.4 (1-16)
Number screened per clinic-enrolled group (mean, range)	33.9 (5-103)	33.3 (3-74)
Number enrolled per clinic-enrolled group (mean, range)	15.8 (1-37)	15.7 (1-37)
Number completing baseline (mITT)-enrolled group (mean, range)	12.8 (1-32)	13.3 (0-29)

© 2026 Dailey DL et al. *JAMA Network Open*.

eTable 3. Results of different imputation methods on movement-evoked pain

Method	Mean ± SD	[95% CI]	p
Observed	-1.18 ± 0.22	[-1.64, -0.72]	<0.0001
Multiple imputation	-1.14 ± 0.22	[-1.58, -0.69]	<0.0001
Imputing Pain at Baseline	-1.02 ± 0.20	[-1.43, -0.61]	<0.0001
Least observation carried forward	-1.03 ± 0.22	[-1.48, -0.57]	<0.0001
Worst observed case	-0.87 ± 0.22	[-1.30, -0.43]	=0.0004
Longitudinal maximum likelihood estimation	-1.13 ± 0.21	[-1.54, -0.71]	<0.0001

© 2026 Dailey DL et al. *JAMA Network Open*.

eTable 4. Sensitivity analysis for those who met the fibromyalgia diagnostic criteria (fibromyalgia-positive) and those who did not meet criteria at baseline (fibromyalgia-negative).

	**Fibromyalgia-negative** n=39 for PT-Only; n=40 for PT+TENS	**Fibromyalgia-Positive** n=131 for PT-Only; n=125 for PT+TENS
Movement-evoked pain (mean, 95%CI)	-1.6 (-2.6, -0.7) p=0.002	-1.0 (-1.5, -0.5) p<0.001
Resting pain (mean, 95%CI)	-1.5 (-2.4, -0.6) p=0.002	-0.8 (-1.4, -0.3) P=0.008
Movement-evoked fatigue (mean, 95% CI)	-1.5, (-2.6, -0.5) p=0.006	-1.1, (-1.7, -0.4) p=0.002
Resting fatigue (mean, 95% CI)	-0.9 (-1.9, 0.1) p=0.09	-0.6 (-1.2, -0.1) p=0.03

© 2026 Dailey DL et al. *JAMA Network Open*.

eTable 5: Sustained Effect of TENS Use in Fibromyalgia*

	PT+TENS (n=191) Randomized + Extension Phases (Day 1-180)	PT-Only (n=193) Extension Phases (Day 60-180)
	**Mean [95% CI]**	**Mean [95% CI]**
Movement-evoked pain (NRS, 0-10)	-0.9 [-1.4, -0.5]	-1.0 [-1.4, -0.6]
Pain at rest (NRS, 0-10)	-0.9 [-1.3, -0.5]	-0.8 [-1.2, -0.4]
Fatigue with movement (NRS, 0-10)	-1.0 [-1.4, -0.5]	-0.9 [-1.3, -0.5]
Fatigue at rest (NRS, 0-10)	-0.9 [-1.3, -0.5]	-0.8 [-1.1, -0.4]
Fibromyalgia impact (FIQR, 0-100)	-7.9 [-10.9, -4.8]	-8.1 [-10.7, -5.6]
Pain severity (BPI, 0-10)	-0.4 [-0.8, -0.1]	-0.6 [-0.9, -0.4]
Pain interference (BPI, 0-10)	-0.9 [-1.3, -0.4]	-0.9 [-1.3, -0.6]
Global Fatigue Index (MAF,1-50)	-4.8 [-6.7, -2.9]	-4.6 [-6.7, -2.6]
Aerobic activity (RAPA 1) (1-7)	-0.1 [-0.3, 0.1]	-0.1 [-0.3, 0.1]
Strength and flexibility (RAPA 2)	0.1 [-0.1, 0.3]	-0.2 [-0.4, -0.0]
Sleep (PROMIS, T-score)	0.7 [-0.1, 1.4]	0.9 [0.3,1.6]
Sleep duration (PROMIS, hrs)	0.1 [-0.2, 0.3]	0.1 [-0.1, 0.3]
Pain catastrophizing (PCS, 0-52)	-3.1 [-4.9, -1.2]	-4.1 (-6.0, -2.2]
Depression (PHQ 8)	-1.0 [-1.8, -0.2]	-1.5 [-2.3, -0.8]
Anxiety (GAD 7)	-0.6 [-1.2, 0.1]	-1.1 [-1.8, -0.3]
Activity average (PSFS, 0 to 10)	1.3 [0.8, 1.8]	0.1 [-0.5, 0.8]
Physical function (PROMIS, T-score)	1.3 [0.8, 1.9]	1.5 [1.0, 2.0]

*The PT-Only group received TENS units with one-on-one telehealth instructions following the randomized phase, *Higher score means worse, †Higher score mean better, MEP: movement-evoked pain, MCID movement pain 1.1 points or 27%; FIQR: Fibromyalgia impact questionnaire revised, MCID 14%[5]; WPI: Widespread pain index; SSS: Symptom severity scale; PSD: Polysymptomatic Distress Scale; BPI: Brief pain inventory; MAF, multidimensional assessment of fatigue; MCID for systemic lupus erythematosus 5.0[6]; NRS: Numeric rating scale, MCID resting pain 1 point or 15%[7]; PROMIS: Patient-reported outcomes measurement information system; PCS: Pain catastrophizing scale, MCID 7.97[8] points; PHQ 8: Personal health questionnaire 8; MCID older adults 5 points[9]; GAD 7: Generalized anxiety disorder 7, MCID 4 points[10]; PSFS: Patient specific functional scale, MCID 2 points[11]; RAPA: Rapid Assessment of Physical Activity

© 2026 Dailey DL et al. *JAMA Network Open*.

eTable 6. Per protocol Analysis*, data are mean with 95% CI

	Mean [95% CI]			
**Outcome**	**Adequate* D1-60 (n=76)**	**Adequate D1-30 (n=30)**	**Inadequate (n=52)**	**PT-Only (n=171)**
Movement-evoked pain	-1.6 [-2.2, -1.0]	-0.9 [-1.6, -0.1]	-0.7 [-1.4, -0.0]	0.0 [-0.3, 0.3]
Pain at rest	-1.1 [-1.7, -0.6]	-0.8 [-1.6, -0.0]	-0.7 [-1.3, -0.0]	-0.1 [-0.4, 0.2]
Fatigue with movement	-1.9 [-2.5, -1.2]	-0.5 [-1.4, 0.3]	-0.6 [-1.3, 0.2]	0.0 [-0.3, 0.3]
Fatigue at rest	-1.2 [-1.8, -0.5]	-0.3 [-1.1, 0.5]	-0.4 [-1.1, 0.3]	-0.1 [-0.4, 0.2]

*Adequate Monthly Dose: 2x/week (8x/month) and 2h/day (total: 180 min/month), Adequate D1-60: Participants used TENS at an adequate dose for Days 1-60, Adequate D1-30: Participants used TENS at an adequate dose for Days 1-30, Inadequate: Participants used TENS less than required for the minimal adequate dose. PT-Only group did not use TENS for the first 60 days. Subject without data=9.

© 2026 Dailey DL et al. *JAMA Network Open*.

eTable 7. Participant experience with TENS

	All<br>n (%)	PT + TENS<br>n (%)	PT-Only<br>n (%)
**Did you find TENS helpful?**			
Yes	217 (81%)	111 (78%)	106 (84%)
No	51 (19%)	31 (22%)	20 (16%)
**How often are you using your TENS unit now?**			
Daily (at least 4 times a week)	147 (55%)	81 (57%)	66 (52%)
At least once a week	66 (25%)	34 (24%)	32 (25%)
At least once a month (less than once a month or not at all)	25 (9%)	10 (7%)	15 (12%)
Other	31 (12%)	18 (13%)	13 (10%)
**If using TENS less than once a month: Why are you not using TENS? (More than 1 may apply)**			
Difficulty putting on electrodes	7 (23%)	6 (33%)	1 (8%)
Feel it's no longer helping	7 (23%)	5 (28%)	2 (15%)
Electrodes irritate the skin	5 (16%)	5 (28%)	0 (0%)
Too hard to wear the unit	4 (13%)	4 (22%)	0 (0%)
TENS unit no longer working	2 (7%)	1 (6%)	1 (8%)

© 2026 Dailey DL et al. *JAMA Network Open*.

eTable 8. Adverse events (AE) related to TENS

Experience (n=358)	Cumulative Number of Experiences (Total)	Number of Safety Participants with Experiences (# part.)	% (#/358)
Anxiety with TENS	22	15	4.2%
Itchiness with TENS	31	22	6.1%
Nausea with TENS	5	4	1.1%
Skin irritation with electrodes	36	24	6.7%
Pain with TENS	35	27	7.5%
Other*	107	72	20.1%
Serious AE-TENS related	0	0	0

*The “other” category primarily involved issues related to operating the TENS unit or the TENS unit not working properly.

© 2026 Dailey DL et al. *JAMA Network Open*.

eTable 9. Adverse events unrelated to TENS

Experiences	Cumulative Number of Experiences (Total)	Number of Safety Participants with Experiences (# part.)	% (#/358)
Hospitalizations	46	41	11.4%
Emergency room visit	112	79	22.1%
Other	1	1	0.3%
Fell during sit and stand test	2	2	0.6%
Fell/Injury during exercise at home	32	25	7.0%

© 2026 Dailey DL et al. *JAMA Network Open*.

eFigure. Per protocol analysis

Bar and scatter plot showing all data points for each group for the per protocol analysis. Bars show the means with standard deviations. A dose response effect was observed in the per protocol analysis showed for change in movement-evoked pain on Day 60 during the randomized phase. An adequate dose of TENS was defined 8x/month and a total of 900 min/mo. Movement-evoked pain was lowest in those who used an adequate dose of TENS for the first 60 days (n=76) followed by those who used an adequate dose for only the first 30 days (n=30). Minimal changes in movement-evoked pain were observed for those who used less than an adequate dose of TENS throughout the 60 day period (n=52). For comparison the group that did not receive TENS (PT-Only group, n=171) showed no change in pain.

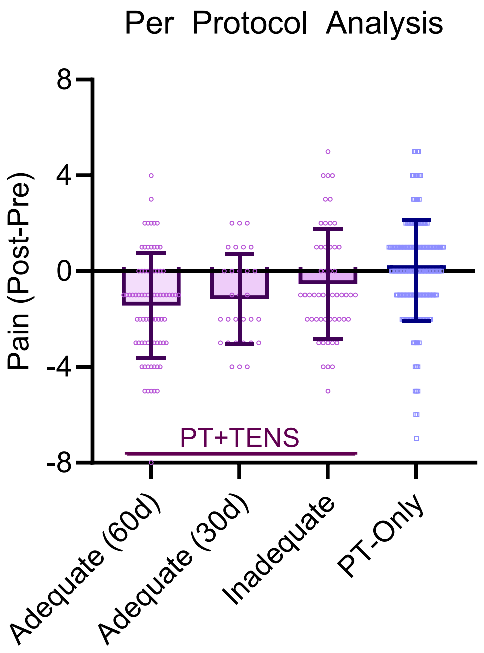

© 2026 Dailey DL et al. *JAMA Network Open*.

eReferences

1. Dailey DL, Vance CGT, Rakel BA, Zimmerman MB, Embree J, Merriwether EN, Geasland KM, Chimenti R, Williams JM, Golchha M, Crofford LJ, Sluka KA. Transcutaneous Electrical Nerve Stimulation Reduces Movement-Evoked Pain and Fatigue: A Randomized, Controlled Trial. *Arthritis Rheumatol*. 2020;72(5):824-836.
2. Vance KG, Pedelty J, Van Gorp BJ, Vance CGT, Johnson EM, Jiang F, Lafontant DE, Koepp M, Post AA, Bayman E, Chimenti RL, Dailey DL, Crofford LJ, Reisinger H, Sluka KA. Community engagement strategies improve recruitment and enrollment in a pragmatic clinical trial. *J Clin Transl Sci*. 2025;9(1):e184.
3. Norman G. Likert scales, levels of measurement and the "laws" of statistics. *Adv Health Sci Educ Theory Pract*. 2010;15(5):625-32.
4. Sullivan GM, Artino AR, Jr. Analyzing and interpreting data from likert-type scales. *J Grad Med Educ*. 2013;5(4):541-2.
5. Bennett RM, Bushmakin AG, Cappelleri JC, Zlateva G, Sadosky AB. Minimal clinically important difference in the fibromyalgia impact questionnaire. *J Rheumatol*. 2009;36(6):1304-11.
6. Goligher EC, Pouchot J, Brant R, Kherani RB, Aviña-Zubieta JA, Lacaille D, Lehman AJ, Ensworth S, Kopec J, Esdaile JM, Liang MH. Minimal clinically important difference for 7 measures of fatigue in patients with systemic lupus erythematosus. *J Rheumatol*. 2008;35(4):635-42.
7. Mease PJ, Spaeth M, Clauw DJ, Arnold LM, Bradley LA, Russell IJ, Kajdasz DK, Walker DJ, Chappell AS. Estimation of minimum clinically important difference for pain in fibromyalgia. *Arthritis Care Res (Hoboken)*. 2011;63(6):821-6.
8. Osman A, Barrios FX, Kopper BA, Hauptmann W, Jones J, O'Neill E. Factor structure, reliability, and validity of the Pain Catastrophizing Scale. *J Behav Med*. 1997;20(6):589-605.
9. Löwe B, Unützer J, Callahan CM, Perkins AJ, Kroenke K. Monitoring depression treatment outcomes with the patient health questionnaire-9. *Med Care*. 2004;42(12):1194-201.
10. Toussaint A, Hüsing P, Gumz A, Wingenfeld K, Härter M, Schramm E, Löwe B. Sensitivity to change and minimal clinically important difference of the 7-item Generalized Anxiety Disorder Questionnaire (GAD-7). *J Affect Disord*. 2020;265:395-401.
11. Stratford P, Gill, C., Westaway, M., Binkley, J. . Assessing disability adn change on individual patients: report of a patient specific measure. *Physiother Can*. 1995;47(4)

© 2026 Dailey DL et al. *JAMA Network Open*.
