# Supplementary material for: Transcutaneous Electrical Nerve Stimulation and Pain With Movement in People With Fibromyalgia: A Cluster Randomized Clinical Trial
Source: JAMA Netw Open. 2026 Mar 27;9(3):e262450. doi: 10.1001/jamanetworkopen.2026.2450 (PMC13032160; doi:10.1001/jamanetworkopen.2026.2450)
Supplement: Supplement 3. — Clinician Group Members [file jamanetwopen-e262450-s003.pdf]

Supplemental Online Content: Nonauthor Collaborators
*First name, last name, and suffix (if applicable) are required and will appear in PubMed.

*Group Name(s): Clinician Group							
***First Name and Middle Initial(s)**	***Last Name**	***Suffix (eg, Jr, III)**	Academic Degrees	Institution	Location (city, state/province, country)	Role or Contribution, eg, chair, principal investigator	Group (if more than 1 Group listed in the byline) and/or Subgroup (eg, Steering Committee)
Cassie	Caldwell		PT, DPT	Kepros Physical Therapy & Performa	Marion, IA, USA	Clinician; recrutiment, enrollment, intervention delivery	
Sharrone	Davis		PT, DPT, SCCE	University of Chicago UI Health	Chicago, IL, USA	Clinician; recrutiment, enrollment, intervention delivery	
Alison	Duncombe		PT, DPT	University of Chicago UI Health	Chicago, IL, USA	Clinician; recrutiment, enrollment, intervention delivery	
Cassie	Flach		PT, DPT	Big Stone Therapies	Baxter, MN, USA	Clinician; recrutiment, enrollment, intervention delivery	
Wade	Fligge		PT, DPT, OCS	Big Stone Therapies	Watertown, SD, USA	Clinician; recrutiment, enrollment, intervention delivery	
Elise	Goodman		PT, DPT	Rock Valley Physical Therapy	Silvis, IL, USA	Clinician; recrutiment, enrollment, intervention delivery	
Casey	Hansen		DPT	Big Stone Therapies	Fargo, ND, USA	Clinician; recrutiment, enrollment, intervention delivery	
Maggie	Ireland		BA	Kepros Physical Therapy & Performa	Cedar Rapids, IA, USA	Clinician; recrutiment, enrollment, intervention delivery	
Rachel	Kiminski		DPT	Big Stone Therapies	Baxter, MN, USA	Clinician; recrutiment, enrollment, intervention delivery	
Ryan	Landberg		DPT	Big Stone Therapies	Marshall, MN, USA	Clinician; recrutiment, enrollment, intervention delivery	
Andrea	Low		PT, DPT, ATC, SCS	Kepros Physical Therapy & Performa	Cedar Rapids, IA, USA	Clinician; recrutiment, enrollment, intervention delivery	
Nicole	Mozafari		PT, DPT, OCS, CMT	Kepros Physical Therapy & Performa	Cedar Rapids, IA, USA	Clinician; recrutiment, enrollment, intervention delivery	
Christopher	O'Connell		PT, DPT, OCS, MTC	Advanced Physical Therapy & Sports	Appleton, WI, USA	Clinician; recrutiment, enrollment, intervention delivery	
Chandler	Riem		DPT, ATC	Advanced Physical Therapy & Sports	Appleton, WI, USA	Clinician; recrutiment, enrollment, intervention delivery	
Anita	Sanchez		PT, DPT	University of Chicago UI Health	Chicago, IL, USA	Clinician; recrutiment, enrollment, intervention delivery	
Kalais	Slaubaugh		PT, DPT	Rock Valley Physical Therapy	Waterloo, IA, USA	Clinician; recrutiment, enrollment, intervention delivery	
Kristina	Tebo		PT, DPT	Advanced Physical Therapy and Wel	Menominee, MI, USA	Clinician; recrutiment, enrollment, intervention delivery	
Kirsten	Verly		DPT	Big Stone Therapies	Marshall, MN, USA	Clinician; recrutiment, enrollment, intervention delivery	
Holly	Wilkinson		MPT	Rock Valley Physical Therapy	Silvis, IL, USA	Clinician; recrutiment, enrollment, intervention delivery	
Adam	Wirtz		DPT	Advanced Physical Therapy Associat	Iola, WI, USA	Clinician; recrutiment, enrollment, intervention delivery	
