# Supplementary material for: Transcutaneous Electrical Nerve Stimulation and Pain With Movement in People With Fibromyalgia: A Cluster Randomized Clinical Trial
Source: JAMA Netw Open. 2026 Mar 27;9(3):e262450. doi: 10.1001/jamanetworkopen.2026.2450 (PMC13032160; doi:10.1001/jamanetworkopen.2026.2450)
Supplement: Supplement 4. — Data Sharing Statement [file jamanetwopen-e262450-s004.pdf]

Data Sharing Statement

Dailey. Transcutaneous Electrical Nerve Stimulation and Pain With Movement in People With Fibromyalgia. *JAMA Netw Open*. Published March 27, 2026.
doi:10.1001/jamanetworkopen.2026.2450

Data
Additional Information: NCT04683042
Data available: Yes
Data types: Deidentified participant data, Data dictionary
How to access data: https://www.icpsr.umich.edu/sites/icpsr/home the Inter-University Consortium for Political and Social Research (ICPSR) repository
When available: With publication

Supporting Documents
Document types: Informed consent form
How to access documents: clinicaltrials.gov
When available: With publication

Additional Information
Who can access the data: anyone requesting data
Types of analyses: any purpose
Mechanisms of data availability: without investigator support
Any additional restrictions: none
